# Human blastocysts of normal and abnormal karyotypes display distinct transcriptome profiles

**DOI:** 10.1038/s41598-018-33279-0

**Published:** 2018-10-08

**Authors:** Frederick Licciardi, Tenzin Lhakhang, Yael G. Kramer, Yutong Zhang, Adriana Heguy, Aristotelis Tsirigos

**Affiliations:** 1 Department of Obstetrics and Gynecology, NYU School of Medicine, New York, 10016 USA; 2 Applied Bioinformatics Laboratories, NYU School of Medicine, New York, 10016 USA; 3 NYU Fertility Center, NYU School of Medicine, New York, 10016 USA; 4 Genome Technology Center, NYU School of Medicine, New York, 10016 USA; 5 Department of Pathology, NYU School of Medicine, New York, 10016 USA; 6 Laura and Isaac Perlmutter Cancer Center and Helen L. and Martin S. Kimmel Center for Stem Cell Biology, NYU School of Medicine, New York, 10016 USA

## Abstract

Unveiling the transcriptome of human blastocysts can provide a wealth of important information regarding early embryonic ontology. Comparing the mRNA production of embryos with normal and abnormal karyotypes allows for a deeper understanding of the protein pathways leading to viability and aberrant fetal development. In addition, identifying transcripts specific for normal or abnormal chromosome copy number could aid in the search for secreted substances that could be used to non-invasively identify embryos best suited for IVF embryo transfer. Using RNA-seq, we characterized the transcriptome of 71 normally developing human blastocysts that were karyotypically normal vs. trisomic or monosomic. Every monosomy and trisomy of the autosomal and sex chromosomes were evaluated, mostly in duplicate. We first mapped the transcriptome of three normal embryos and found that a common core of more than 3,000 genes is expressed in all embryos. These genes represent pathways related to actively dividing cells, such as ribosome biogenesis and function, spliceosome, oxidative phosphorylation, cell cycle and metabolic pathways. We then compared transcriptome profiles of aneuploid embryos to those of normal embryos. We observed that non-viable embryos had a large number of dysregulated genes, some showing a hundred-fold difference in expression. On the contrary, sex chromosome abnormalities, XO and XXX displayed transcriptomes more closely mimicking those embryos with 23 normal chromosome pairs. Intriguingly, we identified a set of commonly deregulated genes in the majority of both trisomies and monosomies. This is the first paper demonstrating a comprehensive transcriptome delineation of karyotypic abnormalities found in the human pre-implantation embryo. We believe that this information will contribute to the development of new pre-implantation genetic screening methods as well as a better understanding of the underlying developmental abnormalities of abnormal embryos, fetuses and children.

## Introduction

The accessibility and popularity of *in vitro* fertilization (IVF) have increased dramatically since the first birth in 1978, such that the process is now responsible for over 2% of children born the USA, 2% in the UK and almost 4% in Japan. Success, as measured by the pregnancy rate per attempt, has sharply increased through enhancements such as optimization of culture conditions^1^, improved rates of oocyte fertilization via intra-cytoplasmic sperm injection^2^, and better rates of sustained implantation using pre-implantation genetic screening (PGS)^3^. Among the animal species, humans are particularly susceptible to embryonic karyotypic abnormalities, which are responsible for the vast majority of implantation failures and miscarriages. While it has been generally understood that increasing age leads to lower fertility and higher miscarriage rates^4^, the IVF/PGS era has more specifically defined this problem of age-related aneuploidy. IVF data has shown that aneuploidy rates increase with maternal age so that by age 30, 30% of embryos are abnormal, 75% by age 40, and 95% by age 44^5^. By helping to select euploidic embryos, PGS has increased the implantation efficiency of each IVF attempt. However, PGS is invasive to the embryo in that it requires breaching of the zonna pellucidia and the manual removal of trophectoderm cells. While the technique has not been shown to increase the rate birth defects or decrease implantation rates^6^, its intrusiveness does raise concerns over minor and yet undetected effects. In addition, the process is labor intensive, requiring significant time allocation of the most experienced embryologists, increasing the overall cost of fertility treatment. A large volume of research is underway to develop non-invasive methods of selecting normal embryos via the analysis of growth by-products. Qualifying and quantifying substances secreted by embryos into their surrounding media would eliminate some of the concerning aspects of embryo biopsy. Metabolomics and proteomics are promising technologies to non-invasively identify targets specific to embryo normalcy^7^; these techniques have yet to reach the appropriate sensitivity levels due to the very small amounts of soluble analytes. The broad spectrum of proteins, both from the embryo and media, further complicate unequivocal detection of relevant molecules. In an effort to more tightly target specific protein markers of embryo normalcy, research has been designed to find differences in the mRNA profiles between karyotypically normal vs abnormal embryos^8^. mRNA lends itself to this type of investigation in that while present is small amounts, copy numbers far exceed those of DNA, and in contrast to proteins, mRNA is infinitely replicable and minimally present in control media. Identifying genes that are highly over or under expressed for each karyotype could greatly narrow down search for protein biomarkers specific to specific karyotype abnormalities. With this in mind, we set out to describe the early embryonic transcriptome of day 5 human blastocysts with all possible mono and trisomies of the autosomal and sex chromosomes. There are other important questions that this research can begin to address. Within each group of specific defects, as is the case for trisomy 21, monosomy X and some others, a small percentage of embryos will escape early pregnancy wastage and develop to birth and beyond, while most other chromosomal abnormalities result in non-viable embryos. The reasons for these outcome differences are unknown. In addition, there is variation in the degree of developmental abnormalities and lifespan among fetuses born within specific abnormal karyotypes. For instance, some children with Down syndrome have cardiac abnormalities but some do not; likewise, mental capacity differs between individuals. Identifying and classifying aberrations in specific developmental pathways may lead to a better understanding of the relationship between alterations in embryonal transcriptomes and specific birth and developmental defects associated with certain abnormal karyotypes.

## Results

### Study Cohort

We analyzed paired-end RNA-sequencing data obtained from normal and aneuploid embryos (Supplementary Table 1). The number of read pairs uniquely aligned to the reference human genome was sufficient to adequately characterize the transcriptome of these embryos: as demonstrated in Supplementary Figure 1A, the number of uniquely aligned read pairs ranged from ~20 to ~40 million. Alignment rates were very high (above 85%) in all samples (Supplementary Figure 1B). Variant calling was performed using the RNA-seq data in order to validate the karyotype of each embryo (see Supplementary Fig. 2 and Methods for details). Out of the 101 embryos in our initial cohort, only 71 were validated using the variant calling approach. Most of the non-validated embryos were found to be mosaic and were excluded from the downstream analysis.

### Characterization of the transcriptome of normal embryos

**Figure 1:**
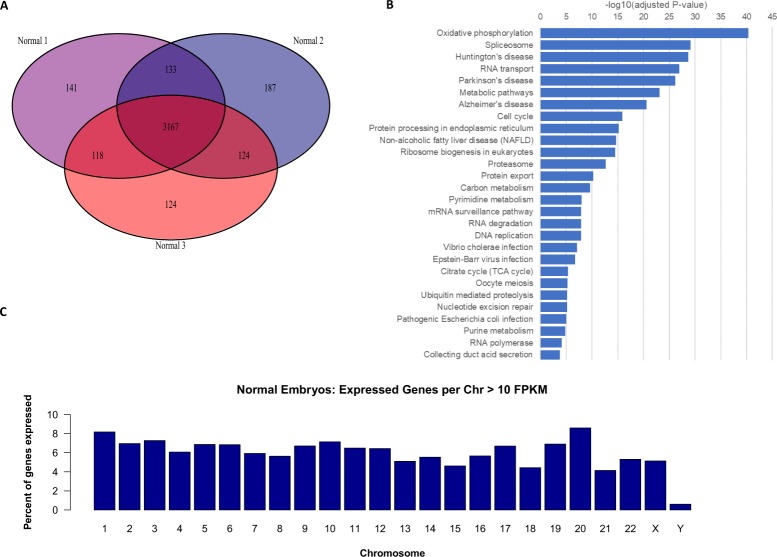
Characterization of the transcriptome of normal embryos. (**A**) Venn diagram of commonly expressed protein-coding genes, (**C**) Venn diagram of commonly expressed protein-coding genes, (**D**) Pathway analysis.

There were three normal, euploid blastocysts in our study cohort. Taking 10 Fragments Per Kilobase of transcript per Million mapped reads (FPKM) as a cutoff for gene expression, we detected a common core of 3,167 transcripts expressed in all three embryos. The overlap of genes expressed by all three embryos was very high, with more than 79% of genes expressed by all three embryos (Fig. 1A). These genes represent pathways related to actively dividing cells such as ribosome biogenesis and function, spliceosome, oxidative phosphorylation, cell cycle, metabolic pathways, and others (Fig. 1B). The complete list of enriched KEGG pathways is made available as Supplementary Table 2. Yan *et al*.^9^ described mRNA production in single cells, from the oocyte to the blastocyst, and in embryonal stem cells (eSCs), but the karyotypes of their specimens were not reported. With the fact that their study involved 30 single cells of the blastocyst, of unknown karyotype, it is difficult to compare their data directly to ours. However, we observed that 1,505 out of the top 2,000 expressed transcripts were common (75%). This overlap is remarkably high especially considering the large technical differences in how the samples were processed and our lack of knowledge of the karyotype of the embryos in the Yan *et al*. study. Two other notable papers describe the transcriptome of human blastocyst single cells. They too make data comparisons difficult due to their lack of control for ploidy status^10,11^. We then calculated the percentage of expressed genes per chromosome, normalized by the number of genes per chromosome (Fig. 1C). The data shows no overrepresentation or underrepresentation of any of the 22 autosomes. Intriguingly, male embryos express some transcripts from the Y chromosome at this early stage, including some protein coding genes and a testis-specific long non-coding RNA. Gene names expressed from chromosome Y across the two male embryos and their average FPKM values are reported in Supplementary Table 3.

### Transcriptional differences between euploid embryos and trisomic embryos

**Figure 2:**
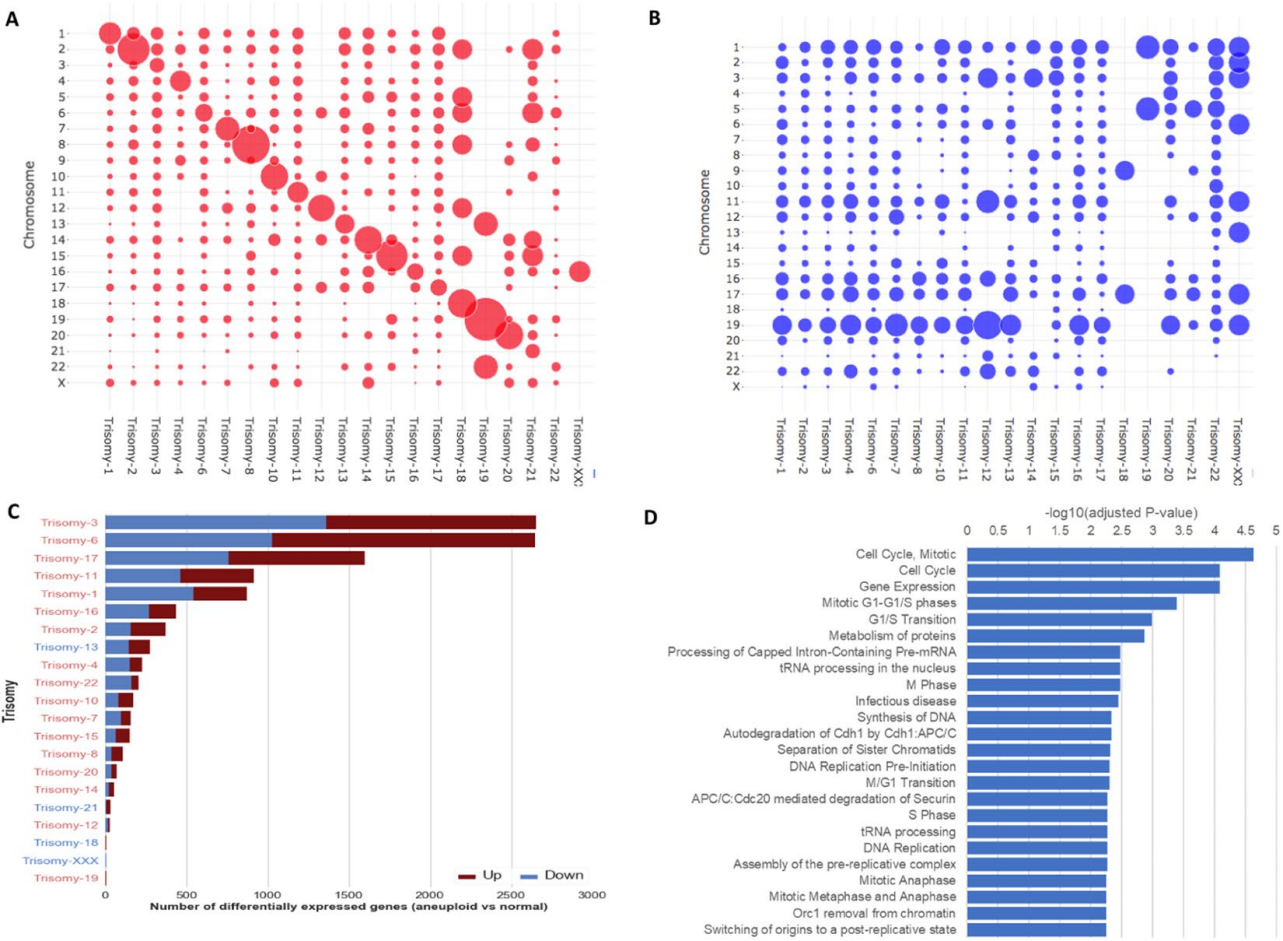
Overview of differential expression analysis of each trisomy type versus normal embryo samples. Bubble-plot representation of the number of (**A**) up-regulated and (**B**) down-regulated genes (trisomy vs normal) per chromosome, normalized by the total number of genes in each chromosome and by the total number of differentially expressed genes in each trisomy, (**C**) Total number of differentially expressed genes per trisomy; trisomies are ordered by the number of differentially expressed genes and labels are colored by viability, (**D**) Enriched or depleted pathways in the sets of differentially expressed genes (per trisomy).

We carried out a differential gene expression analysis to identify transcriptome differences in every trisomy compared to normal blastocysts. In Fig. 2, we provide an overview of the analysis involving all trisomic aneuploidies in our cohort. For the majority of non-viable trisomies, we found large numbers of genes differentially dysregulated when compared to normal embryos. Viable trisomy embryos (trisomy 21; Down syndrome), 18 and XXX (TripleX syndrome) showed few transcriptome changes with respect to normal embryos. In Fig. 2A, we use the bubble-plot representation to show visual overview of the number of up-regulated genes per chromosome and trisomy type. As expected, the largest chromosomal gene up-regulation in each aneuploidy followed a pattern aligned with the respective gain of each chromosome, indicated by the diagonal line. As a negative control, Fig. 2B shows the overview of the number of down-regulated genes per chromosome and trisomy type where we observe no such pattern. In Fig. 2C, we ranked the trisomies by the total number of deregulated genes, placing trisomies with greater gene dysregulation at the top. The majority of the viable trisomy embryos can be associated with fewer gene expression changes. In Fig. 2D, we depict the top pathways that are enriched in genes frequently deregulated in at least four trisomies. All statistically significant pathways (adjusted p-value less than 0.05) are listed in Supplementary Table 4.

### Trisomy 3

**Figure 3:**
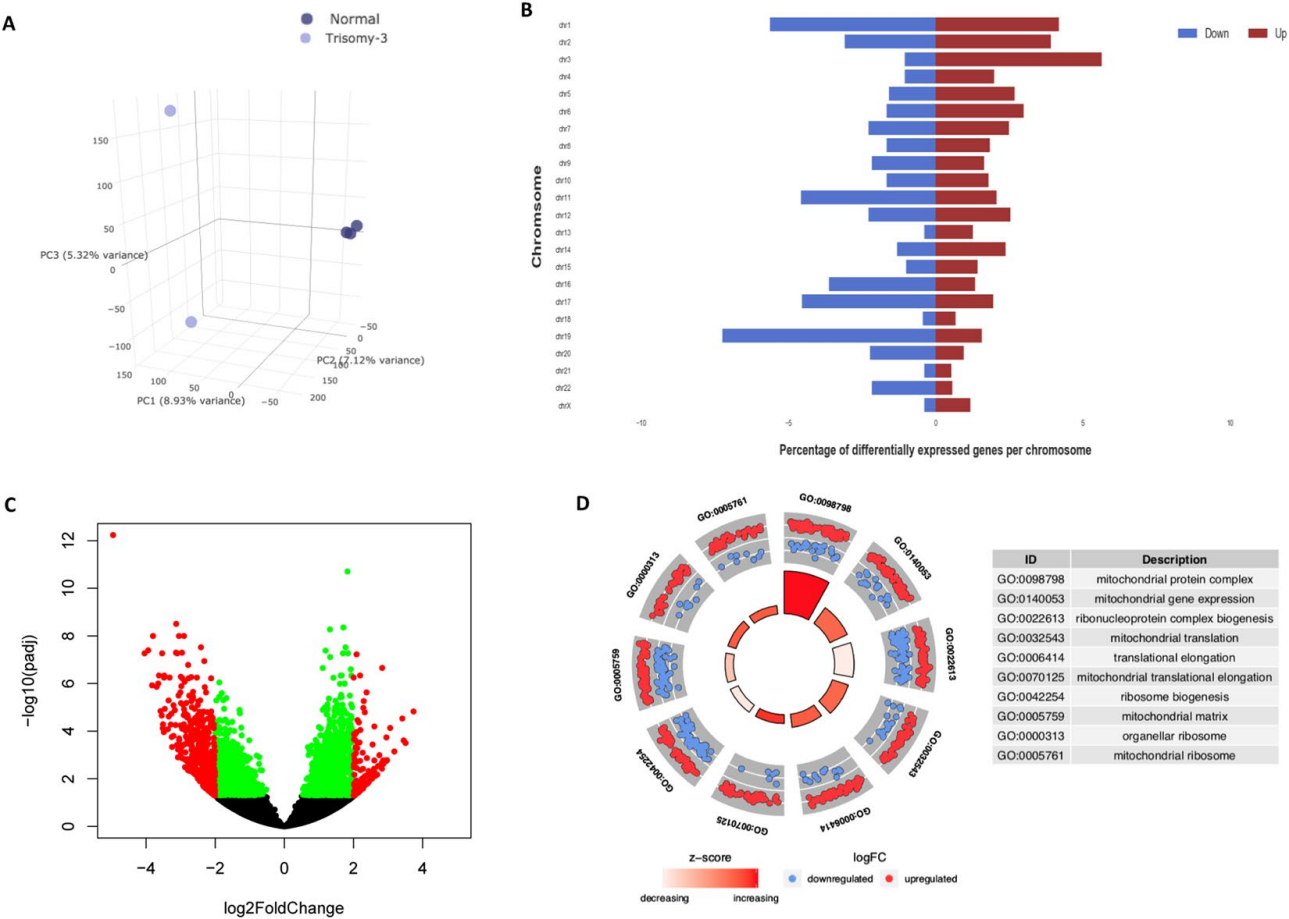
Characterization of Trisomy 3. (**A**) Principle Component Analysis shows separation between aneuploid and normal embryo samples, (**B**) Bar plot displaying the number of genes differentially expressed genes per chromosome, with the red and blue coloring indicating up and down regulation respectively, (**C**) Volcano plot displaying the significant genes color coded by red and green, indicating thresholds of adjusted p-value less than 0.05 and greater than log2 fold-change of 2, (**D**) Top gene ontology terms, significantly enriched in genes that are differentially expressed between trisomic and normal embryo samples.

In Fig. 3, we characterize one of the most dysregulated trisomic embryo, trisomy 3, with more than 2,500 differentially expressed genes. In Fig. 3A we show a 3-D Principal Component Analysis (PCA) of the trisomy 3 embryos along with the normal embryos. The clear separation of the two groups along the first three principle components is evident, indicating that even as early as day 5 post-fertilization, the changes in gene expression caused by this trisomy are substantial. In Fig. 3B we show the number of genes up and down regulated per chromosome using red and blue labels respectively. As expected, we find the genes on chromosome 3 to be the most up-regulated. The volcano plot in Fig. 3C represents the fold-change and adjusted p-values of the dysregulated genes. In Fig. 3D, we show the top gene ontology terms that are enriched in the list of these genes.

### Trisomy 21

We explored the gene expression changes in trisomy 21 blastocysts given that individuals with this aneuploidy survive to adulthood and are often capable of leading normal lives. A detailed list of significantly differentially expressed genes in trisomy 21 embryos is provided in Supplementary Table 5. We found that 35 protein-coding genes were differentially expressed between these embryos and euploid embryos. Remarkably, only 2 of these genes are in chromosome 21. These genes are enriched in antigen presentation/processing and interferon signaling. Also, we noticed the presence of a metallothionein gene (MT1E). Metallothioneins have a neuroprotective role against oxidative stress and neurodegeneration^12^, thus this an interesting finding that suggests these embryos could be under oxidative stress already at this very early stage.

### Transcriptional differences between euploid embryos and monosomic embryos

**Figure 4:**
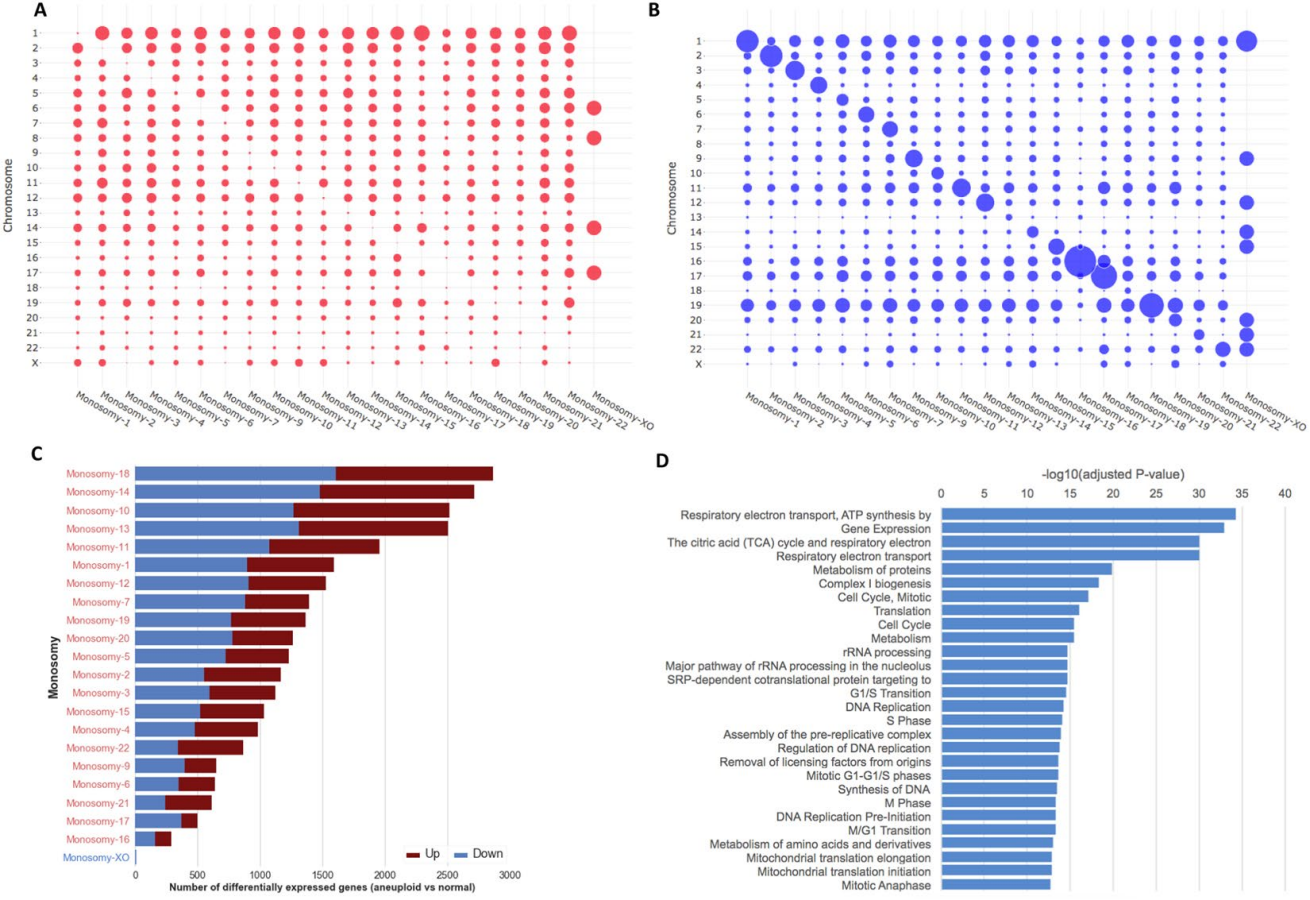
Overview of differential expression analysis of each monosomy type versus normal embryo samples. Bubble-plot representation of the number of (**A**) up-regulated and (**B**) down-regulated genes (monosomy vs normal) per chromosome, normalized by the total number of genes in each chromosome and by the total number of differentially expressed genes in each monosomy, (**C**) Total number of differentially expressed genes per monosomy; monosomies are ordered by the number of differentially expressed genes and labels are colored by viability, (**D**) Enriched or depleted pathways in the sets of differentially expressed genes (per monosomy).

In Fig. 4, we provide an overview of the analysis involving all the monosomies. For most monosomies, we found several differentially expressed genes going in both directions compared to normal embryos (both up- and down-regulated). The number of differentially expressed genes varied by chromosome, even when corrected for per chromosome gene density. In Fig. 4A, we use a bubble-plot representation to provide a quick visual overview of the number of up-regulated genes per chromosome and monosomy type. Similarly, in Fig. 4B, we show the number of down-regulated genes per chromosome and monosomy type. As expected, the largest chromosomal gene down regulation in each aneuploidy followed a pattern similar to that of its respective lost chromosome, indicated by the apparent diagonal line (genes belonging to the lost chromosome tend to appear as down-regulated, as demonstrated by the larger “bubble” size across the diagonal). In Fig. 4C, we rank monosomies by the total number of deregulated genes, placing monosomies that have greater transcriptional changes on top. Gene dysregulation is evident in all autosomal monosomies. In Fig. 4D, we show the top pathways that are enriched in genes frequently deregulated in at least four monosomies. All statistically significant pathways (adjusted p-value less than 0.05) are listed in Supplementary Table 6.

### Monosomy 14

**Figure 5:**
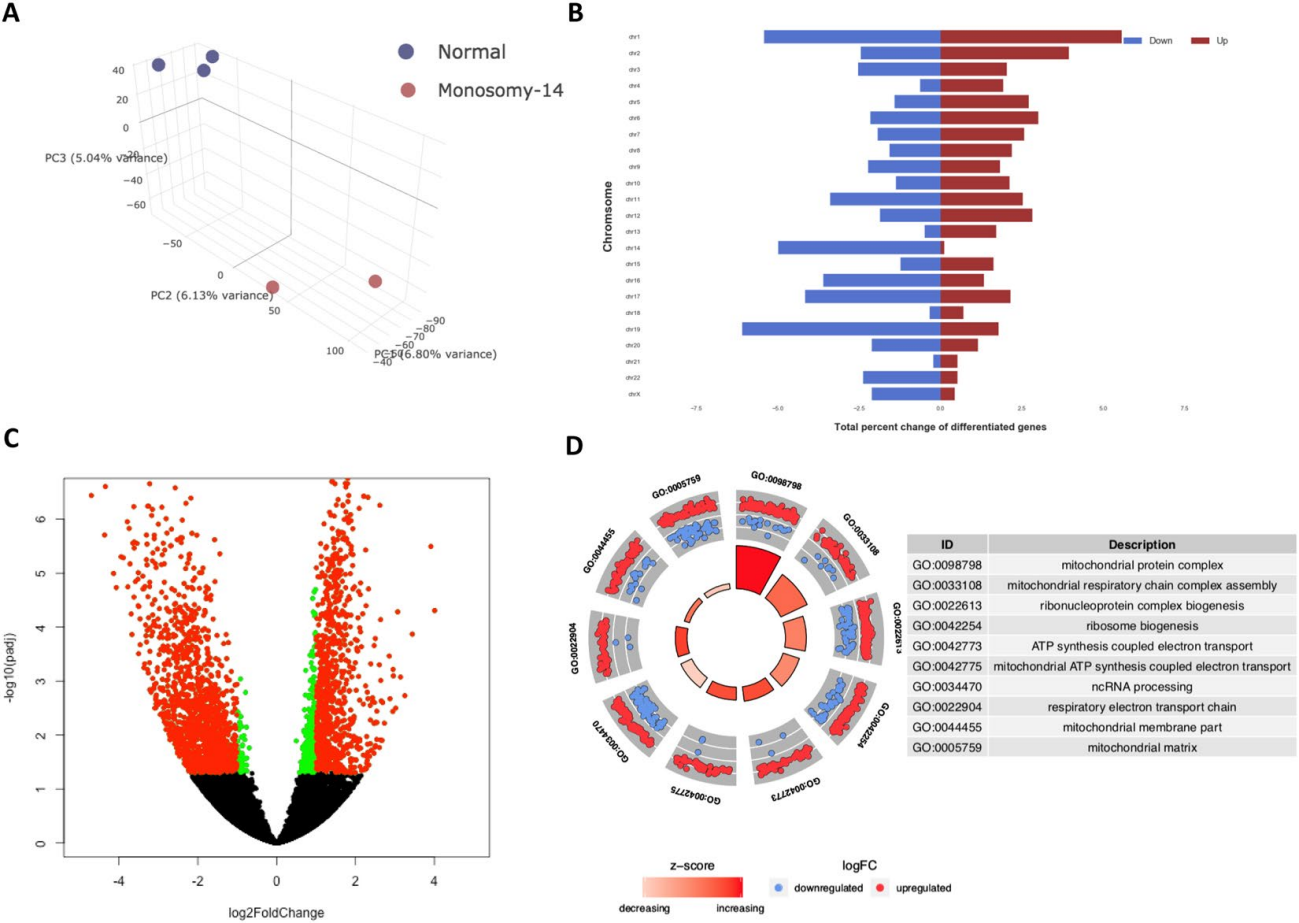
Characterization of Monosomy 14. (**A**) Principle Component Analysis shows separation between aneuploid and normal embryo samples, (**B**) Bar plot displaying the number of genes differentially expressed genes per chromosome, with the red and blue coloring indicating up and down regulation respectively, (**C**) Volcano plot displaying the significant genes color coded by red and green, indicating thresholds of adjusted p-value less than 0.05 and greater than log2 fold-change of 2, (**D**) Top gene ontology terms, significantly enriched in genes that are differentially expressed between monosomic and normal embryo samples.

In Fig. 5, we characterize one of the most dysregulated monosomic embryos, monosomy 14, with more than 2,500 genes differentially expressed. In Fig. 5A we show a 3-D Principal Component Analysis (PCA) of the monosomy 14 embryos along with the normal embryos. The clear separation of the two groups along the first three principle components is evident. In Fig. 5B we show the number of genes up and down regulated per chromosome using red and blue labels respectively. As expected, we find the genes on chromosome 14 to be significantly de-regulated. In Fig. 5C, we show a volcano plot to visually represent the dysregulated genes. In Fig. 5D, we show the top gene ontology terms that are significantly enriched in these genes.

### Frequently deregulated transcripts across aneuploidies

**Figure 6:**
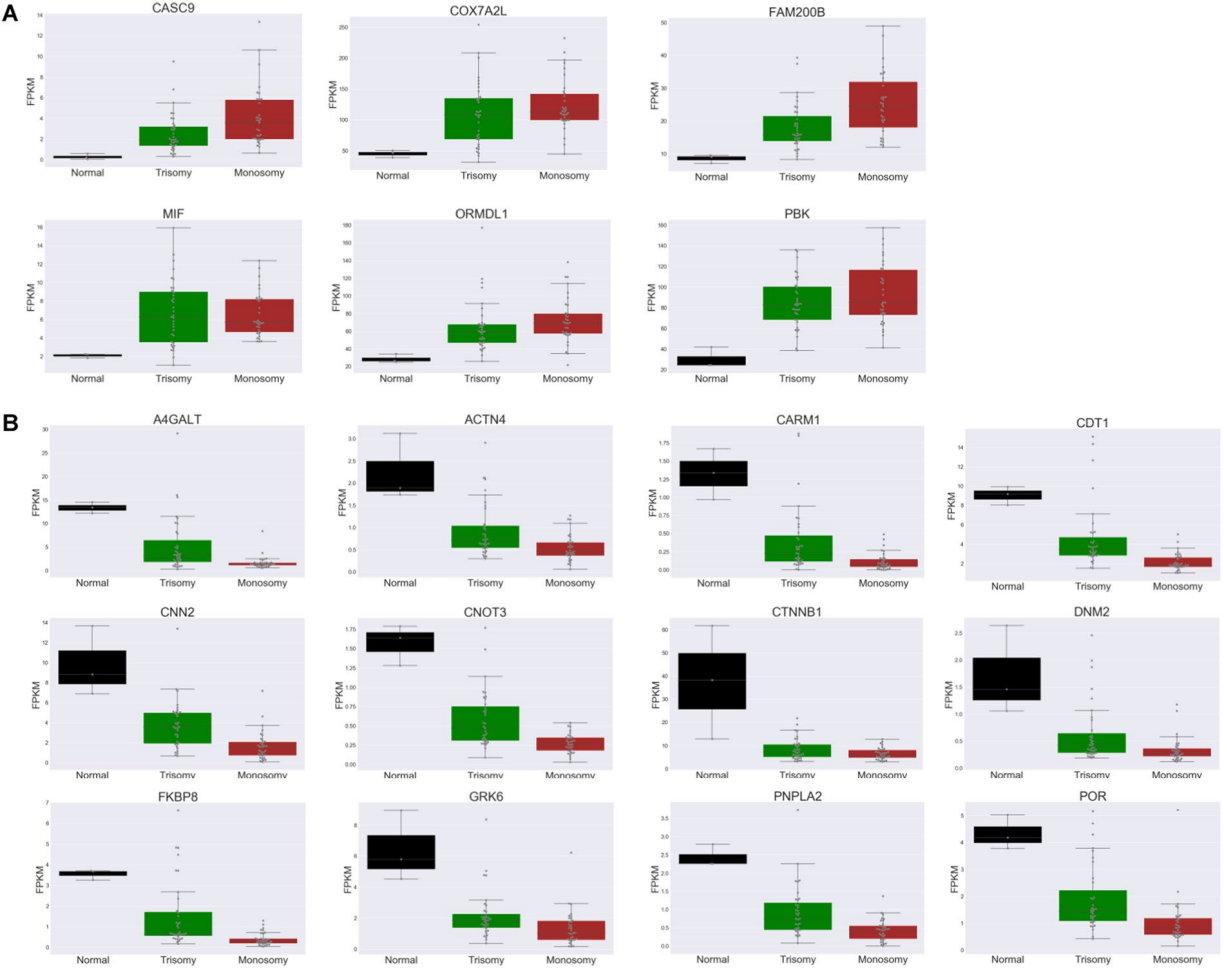
Frequently deregulated genes in trisomies and/or monosomies. Boxplot representation of gene expression levels for commonly up-regulated (**A**) and down-regulated (**B**) genes in both trisomies and monosomies across normal, trisomic and monosomic embryos.

We then investigated whether there are certain transcripts whose expression is consistently deregulated (up- or down-regulated) across many different trisomies and/or monosomies. We focused on aneuploidies with at least 100 differentially expressed genes (12 trisomies and 22 monosomies). Then, for each differentially expressed gene, we calculated the percentage of aneuploidies in which it is found to be up-regulated, down-regulated or unchanged. Then, we selected those genes that change (in either direction) in at least 60% of trisomies or monosomies that had at least 100 differentially expressed genes. Intriguingly, there are several common genes in the two lists. Six genes (CASC9, COX7A2L, FAM200B, MIF, ORMDL1 and PBK) are commonly up-regulated in at least 60% of both trisomies and monosomies. Expression levels of these genes are shown across normal, trisomic and monosomic embryos in the form of boxplots in Fig. 6A. Eighteen genes (A4GALT, ACTN4, CARM1, CDT1, CNN2, CNOT3, CTNNB1, DNM2, FKBP8, GRK6, PNPLA2, POR, RNF10, RNF26, SCYL1, SPINT1, TRPC4AP and ZYX) are down-regulated in at least 60% of the trisomies and monosomies that had at least 100 differentially expressed genes. The expression levels of these genes are shown across normal, trisomic and monosomic embryos in the form of boxplots in Fig. 6B (only 12 out of 18 are shown).

## Discussion

Our study is the first to comprehensively describe the transcriptome of human aneuploidies (trisomies and monosomies) of autosomal and sex chromosomes, and how it differs from euploid (normal) embryos. We also describe the protein pathways that these coding and non-coding genes represent. Unsurprisingly in normal blastocysts, the ~3000 genes expressed at this rapid-growth phase encode proteins in key pathways for actively dividing cells such as ribosome, spliceosome, cell cycle and oxidative phosphorylation. The most striking observation of our study the degree to which embryos with non-viable aneuploidies have massive dysregulation at this early stage, while viable aneuploid embryos do not differ much from euploid blastocysts, suggesting that the non-viability of the majority of the chromosomal abnormalities is caused by very early events in development. Interestingly, the aneuploidy embryos at this stage visibly appear healthy and grow *in vitro* in spite of this vast deregulation indicating that the major metabolic and cell division pathways are not completely affected by these large changes in gene expression. It has been shown that embryos that grow poorly in the laboratory are more like to be aneuploid^13^, however, as verified here, the development of some very anomalous embryos can be indistinguishable from normal. Interestingly, trisomy 21 blastocysts showed very few gene expression changes at this stage, and these changes do not appear to stem predominantly from the extra dosage of chromosome 21 genes. The finding that some of the genes that are induced at this stage play a role in response against oxidative stress suggests that the presence of an extra chromosome 21 somehow results in increased oxidative stress even at this early stage. Oxidative stress has been implicated in the susceptibility of trisomy 21 neurons to oxidative stress^14^. Finding intracellular differences between normal and abnormal embryos could narrow the search for potentially over or under-excreted substances in the culture media. Focusing on mRNA is advantageous because not only might significant differences in intracellular amounts be reflected in differences in extracellular levels, but also because differences could potentially be seen in levels of corresponding proteins, or in the case of trisomy 21 embryos, perhaps increased reactive oxygen species (ROS) can be measured in the culture medium, such as superoxide, peroxyl radical, hydrogen peroxide, hydroxyl radical, and peroxynitrite.

## Conclusions

To our knowledge, this is the first paper demonstrating comprehensive transcriptome delineation of a wide spectrum of karyotypic abnormalities found in the human pre-implantation embryo. We demonstrate that embryos with karyotypes known not to develop past early gestation have more under and over gene expression than those known to deliver at term. Aneuploidy results in wildly altered gene regulation from same-cell normally paired chromosomes. Furthermore, we identified a set of several commonly deregulated genes in the majority of both trisomies and monosomies. Based on this data, we can now shift our focus to characterizing transcripts that are specific to certain chromosomal abnormalities, and we believe that this information will contribute to the development of new PGS methods as well as a better understanding of the underlying developmental abnormalities of abnormal embryos, fetuses and children.

## Methods

### Embryo collection and aneuploidy screening

Research approval was granted by the Institutional Review Board (IRB) of the NYU School of Medicine. Embryos were obtained from patients who had undergone *in vitro* fertilization with PGS for fertility treatment at the New York University Langone Health Fertility Center (NYULFC). Embryos not used for the creation of a pregnancy were donated for research by the patients via written informed consent. In order to control for transcription differences that could result from variations in embryo development, only high-quality blastocysts were included for this project. Embryos that were fragmented, or demonstrated mal-development, premature arrest or nuclear abnormalities were not used. Quality was assessed using the criteria set forth by Gardner *et al*.^15^, which rates the expansion of the blastocyst on a scale of 1–4, 4 being the most advanced. Cellular development of the inner cell mass and trophectoderm are separately rated on a scale of A-D and a-d respectively, with A and a being the most developed. For this project we included embryos considered top quality; those attaining a scale of at least 3Bb. Karyotypic analysis of the initial biopsy procedure was performed via array comparative genomic hybridization (aCGH) or next generation sequencing (NGS). Prior to transcriptome analysis, embryos of known karyotype were thawed, with only embryos that demonstrated at least 90% post thaw cell survival being utilized. A total of 101 supernumerary blastocysts were used for this analysis. Two embryos were independently tested for each abnormality with the exceptions that 5 normals, 5 trisomy 15 s, and three each of trisomy 21,18 and 3 were tested. Only one embryo of trisomy 12 and of monosomies 3, 6 and 12 were available.

### RNA-seq library preparation and sequencing

Embryos were warmed individually via a standard warming protocol for vitrified embryos using an Irvine Scientific Vitrification Thaw kit ®. The expanding blastocyst was then methodically washed in three 50 ul drops of PBS assuring the agglutinative embryo did not contact the bottom of the drop risking adhesion of the embryo to the culture dish. Following washes, the embryo was quickly placed in 9.5ul PBS media and lysed immediately in a microcentrifuge tube, cDNA was synthesized and amplified using the SMARTer v4 Ultra Low kit, with 12 PCR cycles (Clontech, cat #634890), followed by RNA-Seq library preparation (Low Input Library prep, Clontech, cat #6348900, 5 PCR cyles for library amplification. Libraries were sequenced on an Illumina HiSeq2500, as paired end 50 nucleotides in length reads.

### Variant calling and karyotype validation

Sequencing results were demultiplexed and converted to FASTQ format using Illumina bcl2fastq software. The reads were adapter and quality trimmed with Trimmomatic^16^ and then aligned to the human genome (build hg19/GRCh37) using the STAR 2.6 Aligner^17^. Duplicate reads were removed using Sambamba^18^. Further local indel realignment and base-quality score recalibration and are performed using the Genome Analysis Toolkit (GATK 3.6)^19^. Single-nucleotide and small indel somatic variants were called with MuTect2^20^. We then focused on high-confidence variants (sequencing depth at least 50 and quality at least 500), and, for each embryo, we plotted the distribution of observed allele frequencies per chromosome. We then determined the ploidy of each chromosome (haploid, diploid or triploid), by observing the peaks of these distributions: (1) haploid chromosomes are expected to have a single peak near 1, (2) diploids have two peaks at 1/2 and 1, and (3) triploids have three peaks at 1/2, 2/3 and 1.

### RNA-seq alignment, quality assessment and differential expression analysis

The reads were aligned against the hg19 ensemble reference genome/transcriptome utilizing the STAR/2.5 aligner^17^. Subsequent gene counting of each sample was performed utilizing featureCounts/1.5.3^21^. Differential gene expression comparisons based on sex and chromosomal aneuploidy were calculated using DESeq2/3.5, an R Bioconductor package^22^. Genes with an adjusted p-value less than 0.05 were considered to be significantly differentially expressed.

### Pathway analysis

Pathway enrichment analyses were performed using EnrichR^23,24^ on the following gene lists: (1) commonly expressed transcripts of at least 10 FPKM in all five normal embryos, (2) significantly differentially expressed transcripts in at least four trisomies, and, (3) significantly differentially expressed transcripts in at least five monosomies.

### Gene Ontology analysis

Gene Ontology analyses were performed using GOPlot^25^ on the following gene lists: (1) significantly differentially expressed transcripts in trisomy-6 compared to normal embryos, and, (2) significantly differentially expressed transcripts in monosomy-1 compared to normal embryos.

### Ethics approval and consent to participate

Our study itself is conducted as Non-Human Subject Research (since the specimens were all de-identified) and was exempt from IRB review. The collection of specimens themselves on the other hand was conducted under IRB approved study H6902, details below. This IRB approved study allowed us to consent patients interested in allowing us to use their discard material for current studies or store them for future research studies, which we tapped into for the current research: IRB # H6902. Title: Research Studies on Discarded IVF Tissues. Principal Investigator: James A. Grifo, M.D., Ph.D.

Facility: The NYU Fertility Center. All methods were performed in accordance with the relevant guidelines and regulations.

## Electronic supplementary material

Supplementary Information

## Data Availability

Raw data, sample descriptions and processed data (i.e. gene normalized counts across normal, trisomic and monosomic embryos) are made publicly available on GEO, accession number GSE114559.
