# Supplementary material for: Human blastocysts of normal and abnormal karyotypes display distinct transcriptome profiles
Source: Sci Rep. 2018 Oct 8;8:14906. doi: 10.1038/s41598-018-33279-0 (PMC6175822; doi:10.1038/s41598-018-33279-0)
Supplement: Supplementary file 1 — Supplementary Information [file 41598_2018_33279_MOESM1_ESM.pdf]

SUPPLEMENTARY INFORMATION

Human blastocysts of normal and abnormal karyotypes display distinct transcriptome profiles

Frederick Licciardi, Tenzin Lhakhang, Yael G. Kramer, Yutong Zhang, Adriana Heguy and Aristotelis Tsirigos

Supplementary Figure 1. Sequencing and alignment statistics of RNA-seq samples. (A) Number of uniquely aligned read pairs, **(B)** Percentage of uniquely aligned read pairs.

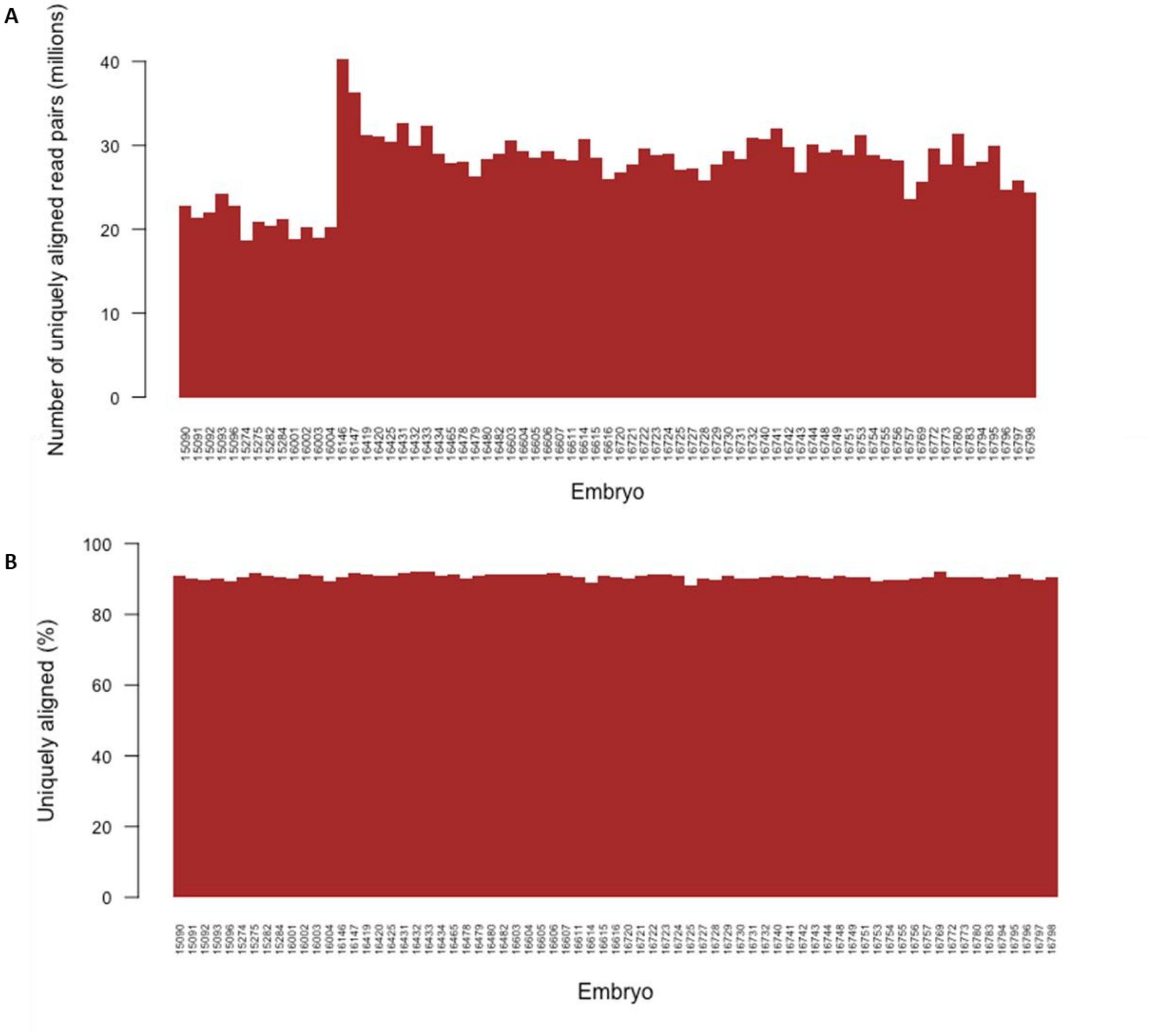

Supplementary Figure 2. Distribution of allele frequencies of detected variants per chromosome for each embryo.

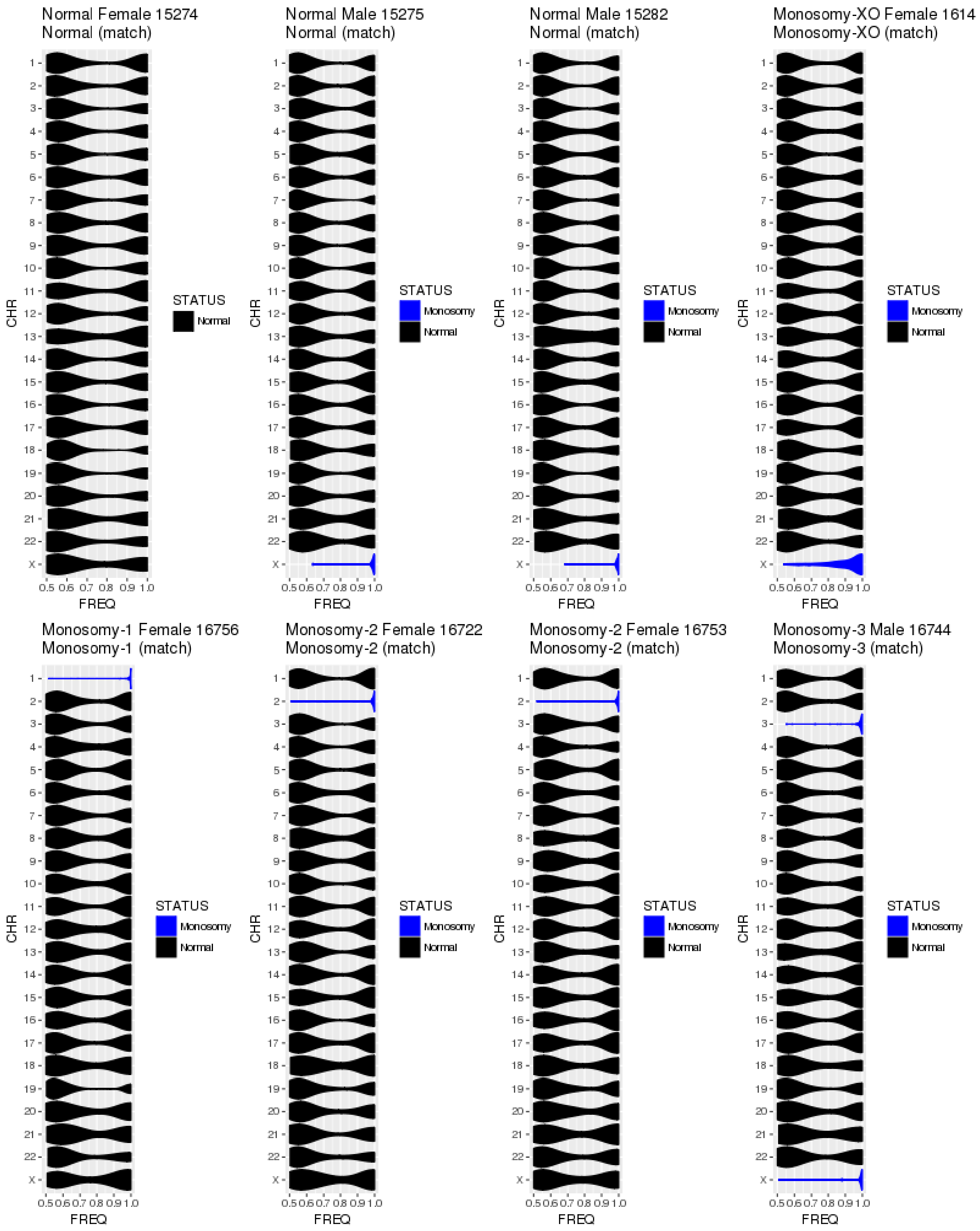

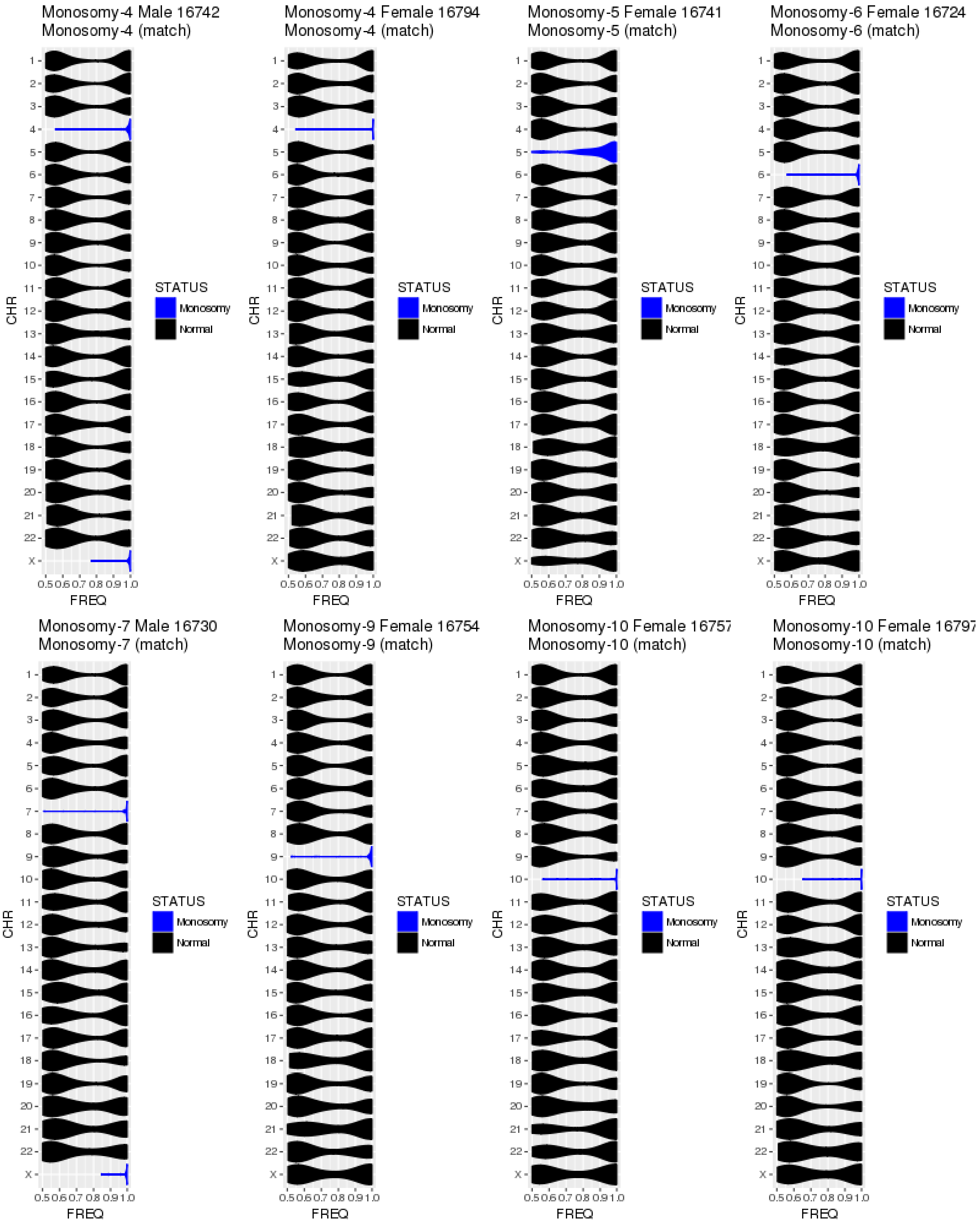

Monosomy-4 Male 16742
Monosomy-4 (match)
Monosomy-4 Female 16794
Monosomy-4 (match)
Monosomy-5 Female 16741
Monosomy-5 (match)
Monosomy-6 Female 16724
Monosomy-6 (match)
Monosomy-7 Male 16730
Monosomy-7 (match)
Monosomy-9 Female 16754
Monosomy-9 (match)
Monosomy-10 Female 16757
Monosomy-10 (match)
Monosomy-10 Female 16797
Monosomy-10 (match)
CHR
FREQ
0.5 0.6 0.7 0.8 0.9 1.0
STATUS
Monosomy
Normal

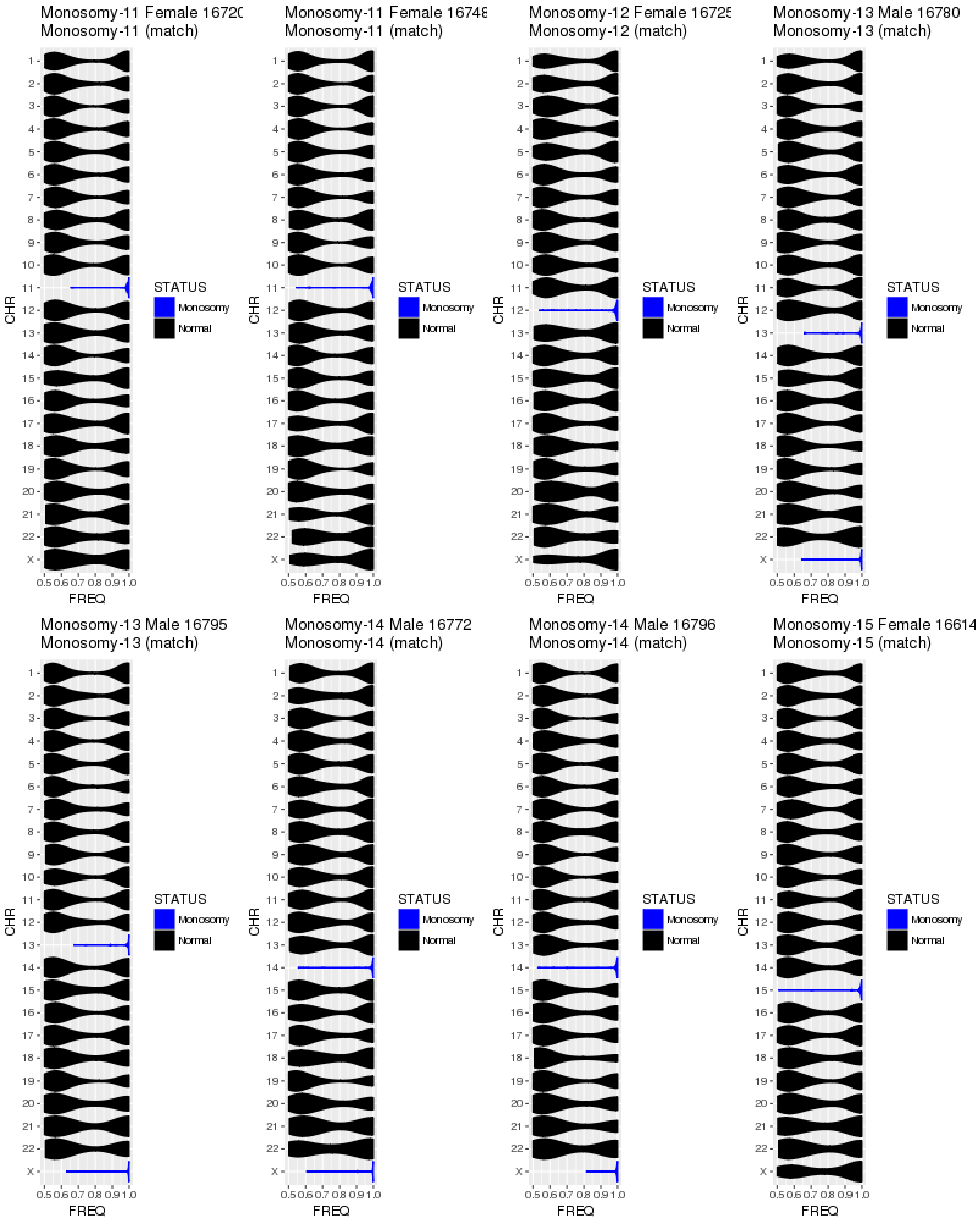

Monosomy-11 Female 16720
Monosomy-11 (match)
Monosomy-11 Female 16748
Monosomy-11 (match)
Monosomy-12 Female 16725
Monosomy-12 (match)
Monosomy-13 Male 16780
Monosomy-13 (match)
Monosomy-13 Male 16795
Monosomy-13 (match)
Monosomy-14 Male 16772
Monosomy-14 (match)
Monosomy-14 Male 16796
Monosomy-14 (match)
Monosomy-15 Female 16614
Monosomy-15 (match)
CHR
1 2 3 4 5 6 7 8 9 10 11 12 13 14 15 16 17 18 19 20 21 22 X
FREQ
0.5 0.6 0.7 0.8 0.9 1.0
STATUS
Monosomy
Normal

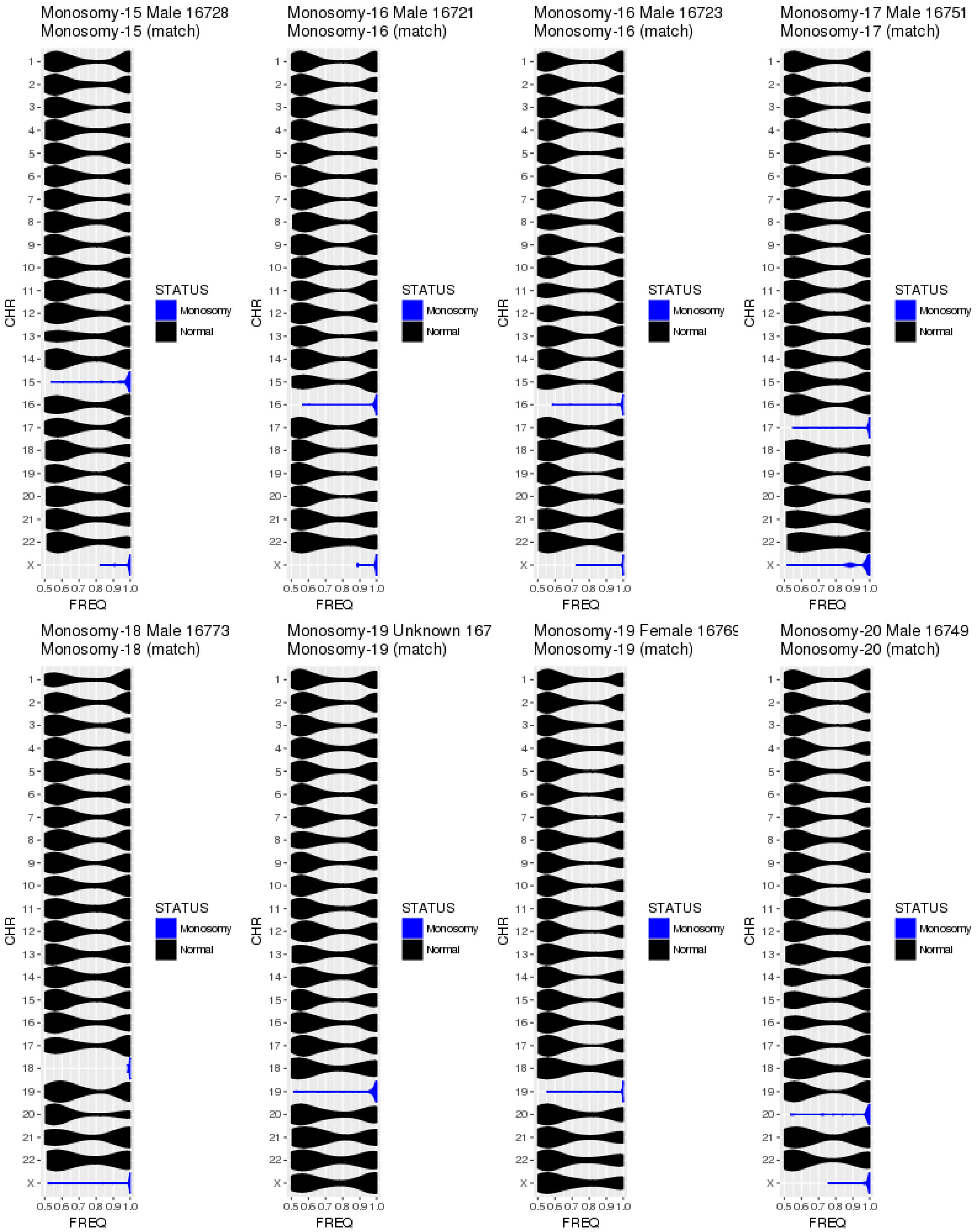
Monosomy-15 Male 16728
Monosomy-15 (match)
Monosomy-16 Male 16721
Monosomy-16 (match)
Monosomy-16 Male 16723
Monosomy-16 (match)
Monosomy-17 Male 16751
Monosomy-17 (match)
Monosomy-18 Male 16773
Monosomy-18 (match)
Monosomy-19 Unknown 167
Monosomy-19 (match)
Monosomy-19 Female 1676
Monosomy-19 (match)
Monosomy-20 Male 16749
Monosomy-20 (match)
CHR
1 2 3 4 5 6 7 8 9 10 11 12 13 14 15 16 17 18 19 20 21 22 X
FREQ
0.5 0.6 0.7 0.8 0.9 1.0
STATUS
Monosomy
Normal

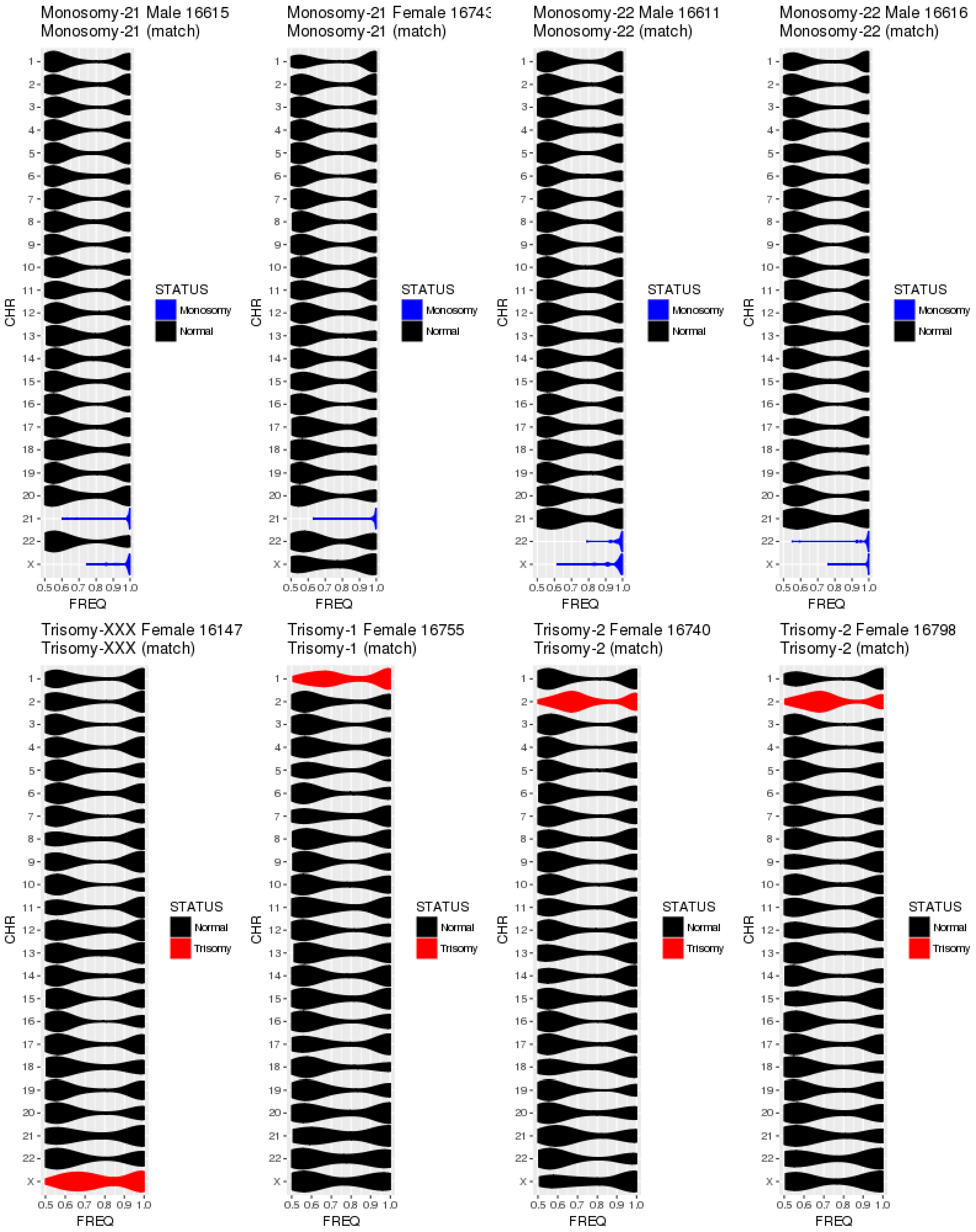

Monosomy-21 Male 16615
Monosomy-21 (match)
Monosomy-21 Female 1674
Monosomy-21 (match)
Monosomy-22 Male 16611
Monosomy-22 (match)
Monosomy-22 Male 16616
Monosomy-22 (match)
Trisomy-XXX Female 16147
Trisomy-XXX (match)
Trisomy-1 Female 16755
Trisomy-1 (match)
Trisomy-2 Female 16740
Trisomy-2 (match)
Trisomy-2 Female 16798
Trisomy-2 (match)
STATUS
Monosomy
Normal
Trisomy
CHR
FREQ
1 2 3 4 5 6 7 8 9 10 11 12 13 14 15 16 17 18 19 20 21 22 X
0.5 0.6 0.7 0.8 0.9 1.0

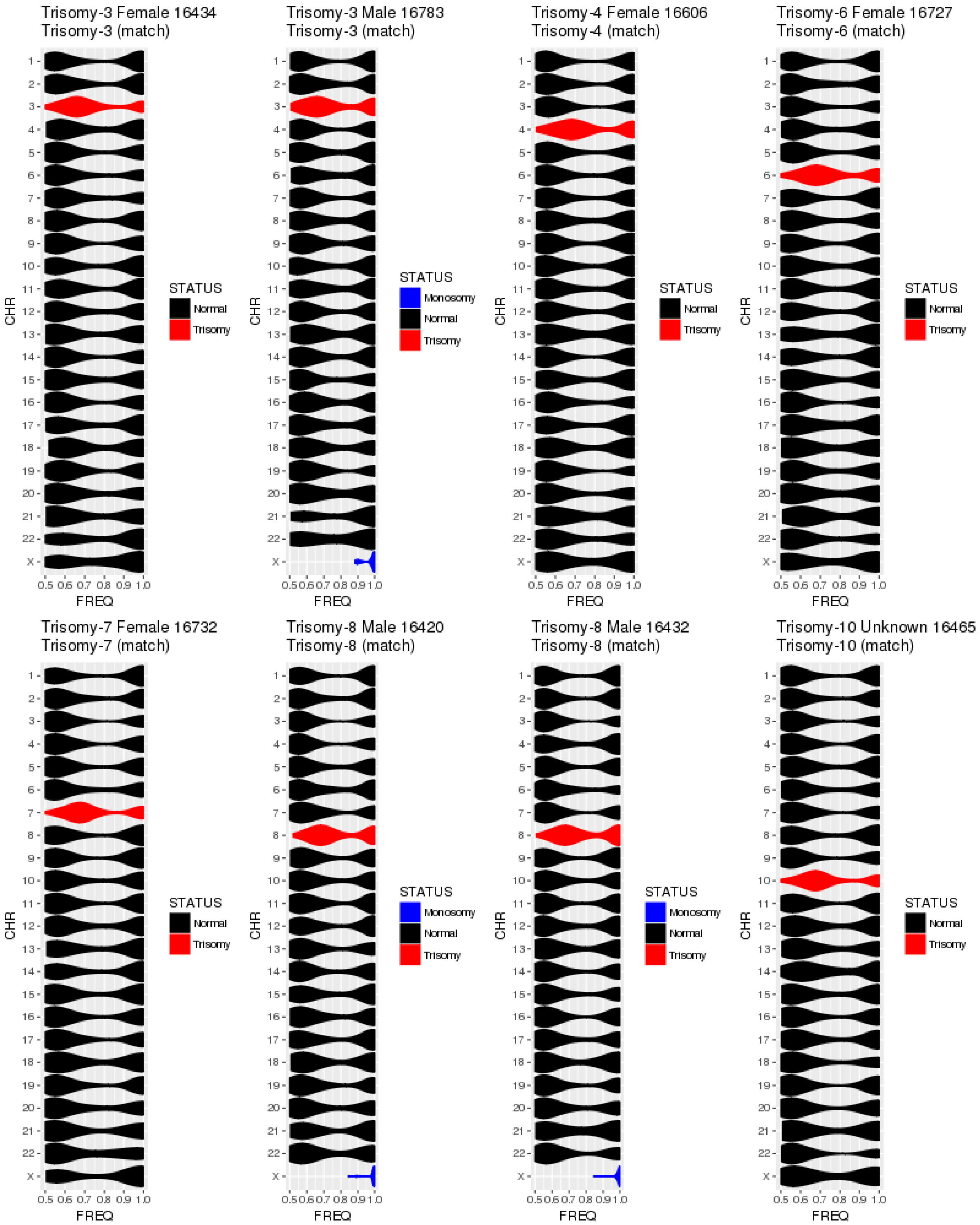
Trisomy-3 Female 16434
Trisomy-3 (match)
Trisomy-3 Male 16783
Trisomy-3 (match)
Trisomy-4 Female 16606
Trisomy-4 (match)
Trisomy-6 Female 16727
Trisomy-6 (match)
Trisomy-7 Female 16732
Trisomy-7 (match)
Trisomy-8 Male 16420
Trisomy-8 (match)
Trisomy-8 Male 16432
Trisomy-8 (match)
Trisomy-10 Unknown 16465
Trisomy-10 (match)
STATUS
Monosomy
Normal
Trisomy
CHR
FREQ
1
2
3
4
5
6
7
8
9
10
11
12
13
14
15
16
17
18
19
20
21
22
X
0.5 0.6 0.7 0.8 0.9 1.0

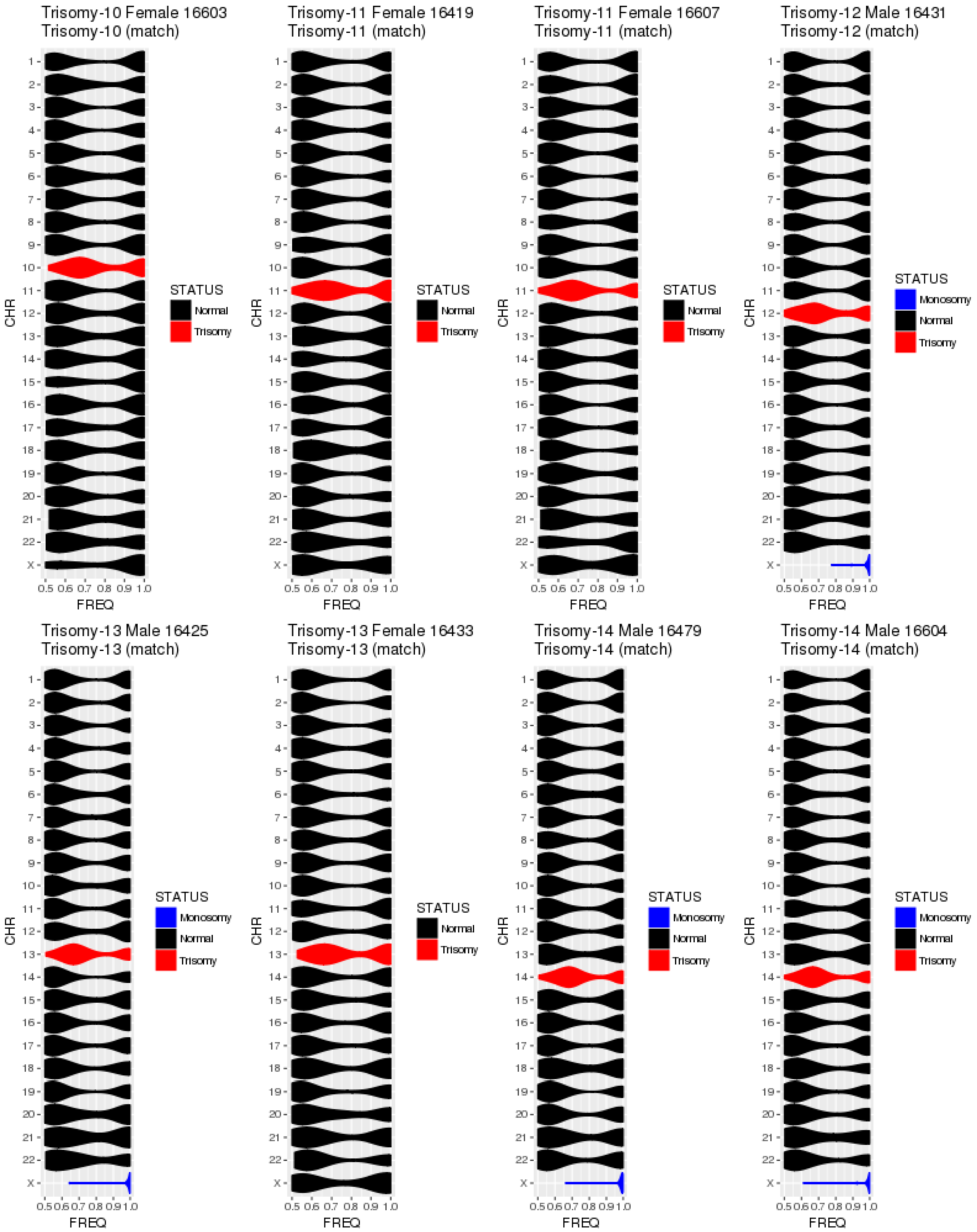
Trisomy-10 Female 16603
Trisomy-10 (match)
Trisomy-11 Female 16419
Trisomy-11 (match)
Trisomy-11 Female 16607
Trisomy-11 (match)
Trisomy-12 Male 16431
Trisomy-12 (match)
Trisomy-13 Male 16425
Trisomy-13 (match)
Trisomy-13 Female 16433
Trisomy-13 (match)
Trisomy-14 Male 16479
Trisomy-14 (match)
Trisomy-14 Male 16604
Trisomy-14 (match)
CHR
1
2
3
4
5
6
7
8
9
10
11
12
13
14
15
16
17
18
19
20
21
22
X
FREQ
0.5
0.6
0.7
0.8
0.9
1.0
STATUS
Monosomy
Normal
Trisomy

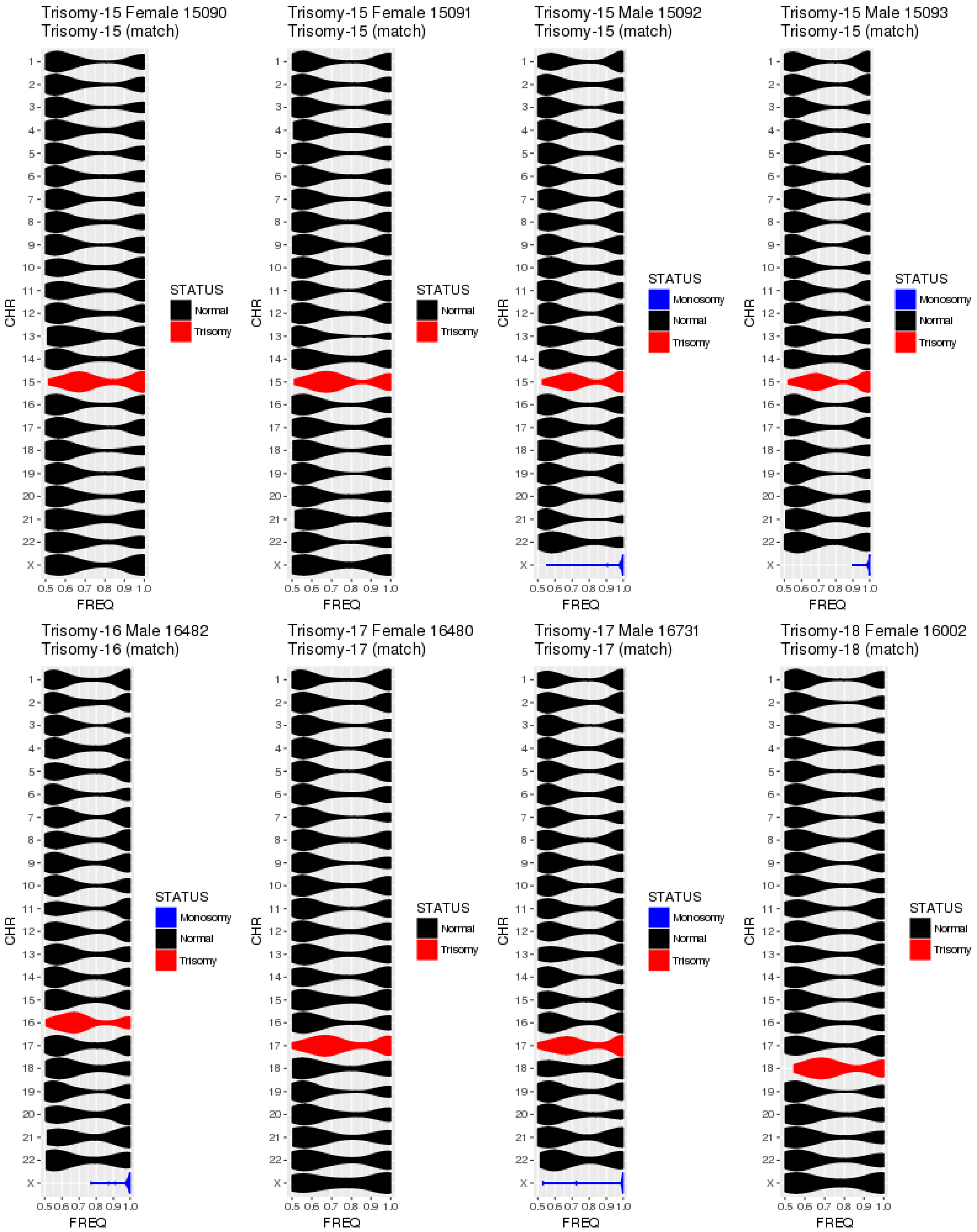

Trisomy-15 Female 15090
Trisomy-15 (match)
STATUS
Normal
Trisomy
CHR
FREQ
Trisomy-15 Female 15091
Trisomy-15 (match)
STATUS
Normal
Trisomy
CHR
FREQ
Trisomy-15 Male 15092
Trisomy-15 (match)
STATUS
Monosomy
Normal
Trisomy
CHR
FREQ
Trisomy-15 Male 15093
Trisomy-15 (match)
STATUS
Monosomy
Normal
Trisomy
CHR
FREQ
Trisomy-16 Male 16482
Trisomy-16 (match)
STATUS
Monosomy
Normal
Trisomy
CHR
FREQ
Trisomy-17 Female 16480
Trisomy-17 (match)
STATUS
Normal
Trisomy
CHR
FREQ
Trisomy-17 Male 16731
Trisomy-17 (match)
STATUS
Monosomy
Normal
Trisomy
CHR
FREQ
Trisomy-18 Female 16002
Trisomy-18 (match)
STATUS
Normal
Trisomy
CHR
FREQ

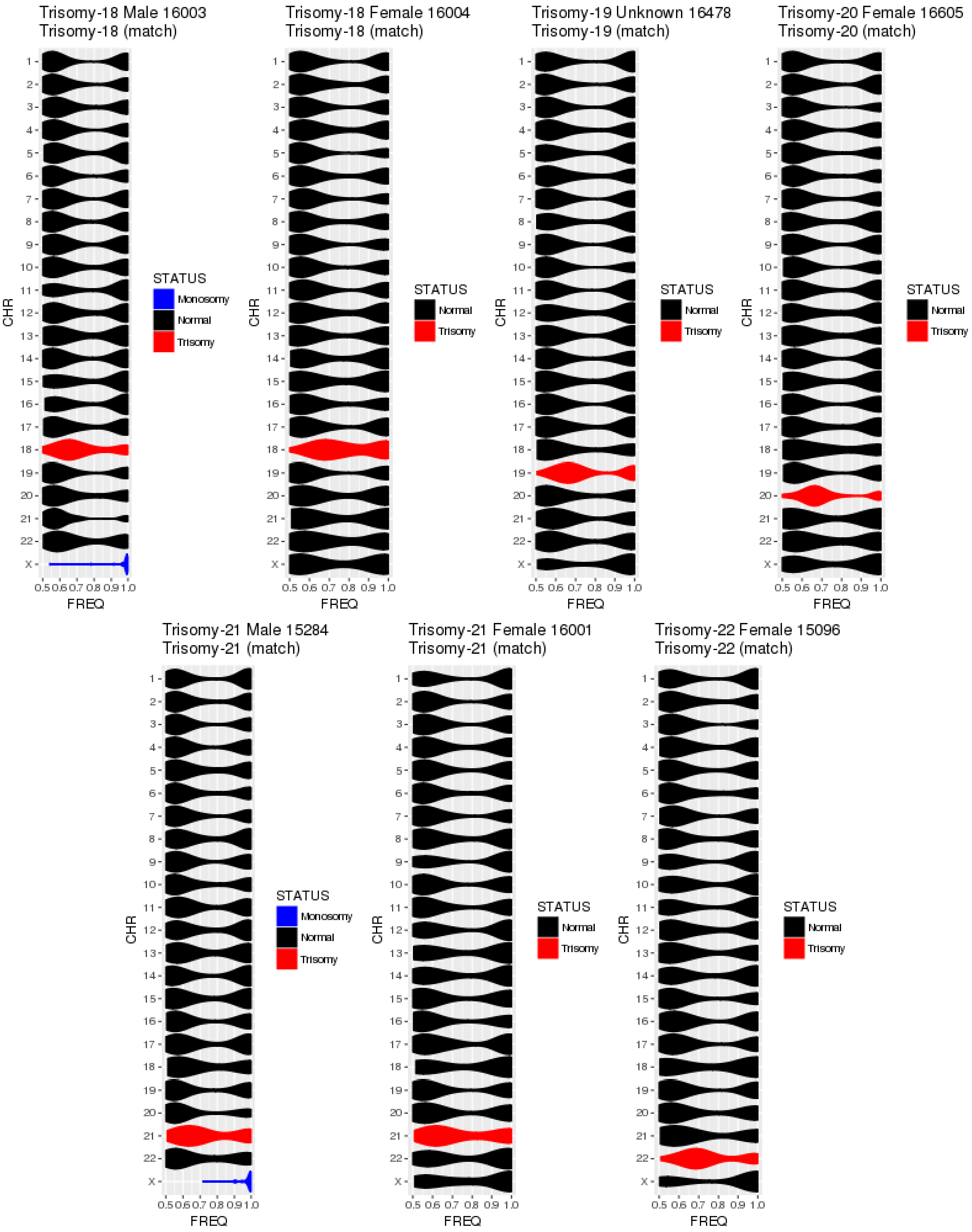
Trisomy-18 Male 16003
Trisomy-18 (match)
STATUS
Monosomy
Normal
Trisomy
Trisomy-18 Female 16004
Trisomy-18 (match)
STATUS
Normal
Trisomy
Trisomy-19 Unknown 16478
Trisomy-19 (match)
STATUS
Normal
Trisomy
Trisomy-20 Female 16605
Trisomy-20 (match)
STATUS
Normal
Trisomy
Trisomy-21 Male 15284
Trisomy-21 (match)
STATUS
Monosomy
Normal
Trisomy
Trisomy-21 Female 16001
Trisomy-21 (match)
STATUS
Normal
Trisomy
Trisomy-22 Female 15096
Trisomy-22 (match)
STATUS
Normal
Trisomy
CHR
1
2
3
4
5
6
7
8
9
10
11
12
13
14
15
16
17
18
19
20
21
22
X
FREQ
0.5 0.6 0.7 0.8 0.9 1.0

Supplementary Table 1. Dataset description. List of embryos used in this study with sex and karyotype information.

SAMPLE ID	SEX	STATUS
15274	Female	Normal
15275	Male	Normal
15282	Male	Normal
16146	Female	Monosomy-XO
16756	Female	Monosomy-1
16722	Female	Monosomy-2
16753	Female	Monosomy-2
16744	Male	Monosomy-3
16742	Male	Monosomy-4
16794	Female	Monosomy-4
16741	Female	Monosomy-5
16724	Female	Monosomy-6
16730	Male	Monosomy-7
16754	Female	Monosomy-9
16757	Female	Monosomy-10
16797	Female	Monosomy-10
16720	Female	Monosomy-11
16748	Female	Monosomy-11
16725	Female	Monosomy-12
16780	Male	Monosomy-13
16795	Male	Monosomy-13
16772	Male	Monosomy-14
16796	Male	Monosomy-14

16614	Female	Monosomy-15
16728	Male	Monosomy-15
16721	Male	Monosomy-16
16723	Male	Monosomy-16
16751	Male	Monosomy-17
16773	Male	Monosomy-18
16729	Unknown	Monosomy-19
16769	Female	Monosomy-19
16749	Male	Monosomy-20
16615	Male	Monosomy-21
16743	Female	Monosomy-21
16611	Male	Monosomy-22
16616	Male	Monosomy-22
16147	Female	Trisomy-XXX
16755	Female	Trisomy-1
16740	Female	Trisomy-2
16798	Female	Trisomy-2
16434	Female	Trisomy-3
16783	Male	Trisomy-3
16606	Female	Trisomy-4
16727	Female	Trisomy-6
16732	Female	Trisomy-7
16420	Male	Trisomy-8
16432	Male	Trisomy-8
16465	Unknown	Trisomy-10
16603	Female	Trisomy-10
16419	Female	Trisomy-11
16607	Female	Trisomy-11
16431	Male	Trisomy-12
16425	Male	Trisomy-13
16433	Female	Trisomy-13
16479	Male	Trisomy-14
16604	Male	Trisomy-14
15090	Female	Trisomy-15

15091	Female	Trisomy-15
15092	Male	Trisomy-15
15093	Male	Trisomy-15
16482	Male	Trisomy-16
16480	Female	Trisomy-17
16731	Male	Trisomy-17
16002	Female	Trisomy-18
16003	Male	Trisomy-18
16004	Female	Trisomy-18
16478	Unknown	Trisomy-19
16605	Female	Trisomy-20
15284	Male	Trisomy-21
16001	Female	Trisomy-21
15096	Female	Trisomy-22

Supplementary Table 2. List of enriched KEGG pathways. P-values, adjusted P-values and enrichment scores of significantly enriched KEGG pathways in the list of commonly expressed genes across five normal embryos.

Term	P-value	Adjusted P-value
Oxidative phosphorylation_Homo sapiens_hsa00190	3.0681E-43	4.2953E-41
Spliceosome_Homo sapiens_hsa03040	7.6073E-32	7.1002E-30
Huntington's disease_Homo sapiens_hsa05016	3.1625E-31	2.2137E-29
RNA transport_Homo sapiens_hsa03013	1.9605E-29	1.0979E-27
Parkinson's disease_Homo sapiens_hsa05012	1.6323E-28	7.6173E-27
Metabolic pathways_Homo sapiens_hsa01100	1.9051E-25	7.6205E-24
Alzheimer's disease_Homo sapiens_hsa05010	6.8313E-23	2.391E-21
Cell cycle_Homo sapiens_hsa04110	3.9329E-18	1.2236E-16
Protein processing in endoplasmic reticulum_Homo sapiens_hsa04141	2.1001E-17	5.8803E-16
Non-alcoholic fatty liver disease (NAFLD)_Homo sapiens_hsa04932	8.1098E-17	2.0643E-15
Ribosome biogenesis in eukaryotes_Homo sapiens_hsa03008	1.2539E-16	2.9257E-15
Proteasome_Homo sapiens_hsa03050	1.0021E-14	2.1584E-13
Protein export_Homo sapiens_hsa03060	2.7618E-12	5.5236E-11
Carbon metabolism_Homo sapiens_hsa01200	1.2837E-11	2.3962E-10
Pyrimidine metabolism_Homo sapiens_hsa00240	5.9815E-10	1.0468E-08
mRNA surveillance pathway_Homo sapiens_hsa03015	7.6247E-10	1.2558E-08
RNA degradation_Homo sapiens_hsa03018	8.1357E-10	1.2656E-08
DNA replication_Homo sapiens_hsa03030	9.053E-10	1.3341E-08
Vibrio cholerae infection_Homo sapiens_hsa05110	5.9175E-09	8.2845E-08
Epstein-Barr virus infection_Homo sapiens_hsa05169	1.3952E-08	1.8602E-07
Citrate cycle (TCA cycle)_Homo sapiens_hsa00020	3.5576E-07	4.5278E-06
Oocyte meiosis_Homo sapiens_hsa04114	4.3829E-07	5.3357E-06
Ubiquitin mediated proteolysis_Homo sapiens_hsa04120	6.0624E-07	6.7899E-06
Nucleotide excision repair_Homo sapiens_hsa03420	5.9454E-07	6.7899E-06
Pathogenic Escherichia coli infection_Homo sapiens_hsa05130	9.1786E-07	9.8846E-06
Purine metabolism_Homo sapiens_hsa00230	1.3861E-06	1.4374E-05
RNA polymerase_Homo sapiens_hsa03020	7.0806E-06	7.0806E-05
Collecting duct acid secretion_Homo sapiens_hsa04966	1.5608E-05	0.0001507
Lysosome_Homo sapiens_hsa04142	2.165E-05	0.00019635
Epithelial cell signaling in Helicobacter pylori infection_Homo sapiens_hsa05120	2.1739E-05	0.00019635
Terpenoid backbone biosynthesis_Homo sapiens_hsa00900	3.3066E-05	0.00028933
Biosynthesis of amino acids_Homo sapiens_hsa01230	3.7492E-05	0.00031811
Synaptic vesicle cycle_Homo sapiens_hsa04721	5.2068E-05	0.0004288
Mismatch repair_Homo sapiens_hsa03430	5.917E-05	0.00047336
Basal transcription factors_Homo sapiens_hsa03022	8.5013E-05	0.00064334
Cysteine and methionine metabolism_Homo sapiens_hsa00270	8.5013E-05	0.00064334
Shigellosis_Homo sapiens_hsa05131	9.1172E-05	0.00067179
Cardiac muscle contraction_Homo sapiens_hsa04260	0.00010347	0.00074283
Aminoacyl-tRNA biosynthesis_Homo sapiens_hsa00970	0.00011915	0.00083408

Phagosome_Homo sapiens_hsa04145	0.00041242	0.00281653
Endocytosis_Homo sapiens_hsa04144	0.00043036	0.00283498
Glycolysis / Gluconeogenesis_Homo sapiens_hsa00010	0.00043537	0.00283498
Propanoate metabolism_Homo sapiens_hsa00640	0.00068037	0.00432965
Valine, leucine and isoleucine degradation_Homo sapiens_hsa00280	0.00072113	0.004487
Pentose phosphate pathway_Homo sapiens_hsa00030	0.00089629	0.00545568
Base excision repair_Homo sapiens_hsa03410	0.00096124	0.00572655
Glutathione metabolism_Homo sapiens_hsa00480	0.00198635	0.01158703
Fatty acid metabolism_Homo sapiens_hsa01212	0.00212657	0.01215184
Pyruvate metabolism_Homo sapiens_hsa00620	0.00236281	0.01323173
2-Oxocarboxylic acid metabolism_Homo sapiens_hsa01210	0.00247875	0.0136088
Fatty acid elongation_Homo sapiens_hsa00062	0.00322284	0.01735378
Bacterial invasion of epithelial cells_Homo sapiens_hsa05100	0.00394277	0.02082971
Viral carcinogenesis_Homo sapiens_hsa05203	0.00485955	0.02519765
N-Glycan biosynthesis_Homo sapiens_hsa00510	0.0071061	0.03617649
Glyoxylate and dicarboxylate metabolism_Homo sapiens_hsa00630	0.00823326	0.04050666
Steroid biosynthesis_Homo sapiens_hsa00100	0.008246	0.04050666
p53 signaling pathway_Homo sapiens_hsa04115	0.00930613	0.04492613

Supplementary Table 3. List of expressed chromosome Y genes. Gene symbols, gene descriptions and average FPKM values of chromosome Y genes (cutoff FPKM = 1).

Gene Symbol	Mean	Gene Biotype	Description
RPS4Y1	92.00	protein_coding	ribosomal protein S4, Y-linked 1 [Source:HGNC Symbol;Acc:HGNC:10425]
CD24P4	41.92	processed_pseudogene	CD24 molecule pseudogene 4 [Source:HGNC Symbol;Acc:HGNC:1649]
EIF1AY	18.78	protein_coding	eukaryotic translation initiation factor 1A, Y-linked [Source:HGNC Symbol;Acc:HGNC:3252]
DDX3Y	8.55	protein_coding	DEAD (Asp-Glu-Ala-Asp) box helicase 3, Y-linked [Source:HGNC Symbol;Acc:HGNC:2699]
PSMA6P1	2.64	processed_pseudogene	proteasome (prosome, macropain) subunit, alpha type, 6 pseudogene 1 [Source:HGNC Symbol;Acc:HGNC:17460]
ZFY	2.13	protein_coding	zinc finger protein, Y-linked [Source:HGNC Symbol;Acc:HGNC:12870]
RNA5-8SP6	1.78	rRNA	RNA, 5.8S ribosomal pseudogene 6 [Source:HGNC Symbol;Acc:HGNC:41960]
AC010970.2	1.03	processed_pseudogene	N/A
TTTY15	1.02	lincRNA	testis-specific transcript, Y-linked 15 (non-protein coding) [Source:HGNC Symbol;Acc:HGNC:18567]

Supplementary Table 4. List of enriched pathways (trisomies). P-values, adjusted P-values and enrichment scores of significantly enriched pathways in the list of genes that are frequently deregulated in trisomies.

Term	P-value	Adjusted P-value
Cell Cycle, Mitotic_Homo sapiens_R-HSA-69278	2.6512E-08	2.3251E-05
HIV Infection_Homo sapiens_R-HSA-162906	1.9092E-07	8.1589E-05
Cell Cycle_Homo sapiens_R-HSA-1640170	2.9542E-07	8.1589E-05
Gene Expression_Homo sapiens_R-HSA-74160	3.7213E-07	8.1589E-05
HIV Life Cycle_Homo sapiens_R-HSA-162587	8.0225E-07	0.00014071
Mitotic G1-G1/S phases_Homo sapiens_R-HSA-453279	2.7651E-06	0.00040417
Late Phase of HIV Life Cycle_Homo sapiens_R-HSA-162599	7.2631E-06	0.00090997
G1/S Transition_Homo sapiens_R-HSA-69206	9.4001E-06	0.00103048
Metabolism of proteins_Homo sapiens_R-HSA-392499	1.3816E-05	0.00134631
Processing of Capped Intron-Containing Pre-mRNA_Homo sapiens_R-HSA-72203	3.7575E-05	0.00329537
tRNA processing in the nucleus_Homo sapiens_R-HSA-6784531	4.1958E-05	0.00334517
M Phase_Homo sapiens_R-HSA-68886	4.5837E-05	0.00334995
Infectious disease_Homo sapiens_R-HSA-5663205	5.2829E-05	0.00356394
Synthesis of DNA_Homo sapiens_R-HSA-69239	7.2343E-05	0.00453177
Autodegradation of Cdh1 by Cdh1:APC/C_Homo sapiens_R-HSA-174084	7.7746E-05	0.00454557
Separation of Sister Chromatids_Homo sapiens_R-HSA-2467813	8.8529E-05	0.00485248
DNA Replication Pre-Initiation_Homo sapiens_R-HSA-69002	0.00010165	0.0049526
M/G1 Transition_Homo sapiens_R-HSA-68874	0.00010165	0.0049526
APC/C:Cdc20 mediated degradation of Securin_Homo sapiens_R-HSA-174154	0.00012201	0.00526204
S Phase_Homo sapiens_R-HSA-69242	0.000126	0.00526204
tRNA processing_Homo sapiens_R-HSA-72306	0.00012024	0.00526204
DNA Replication_Homo sapiens_R-HSA-69306	0.00014123	0.00538508
Assembly of the pre-replicative complex_Homo sapiens_R-HSA-68867	0.00013584	0.00538508
Mitotic Anaphase_Homo sapiens_R-HSA-68882	0.00017148	0.00560296
Mitotic Metaphase and Anaphase_Homo sapiens_R-HSA-2555396	0.00018157	0.00560296
Host Interactions of HIV factors_Homo sapiens_R-HSA-162909	0.00017911	0.00560296
Orc1 removal from chromatin_Homo sapiens_R-HSA-68949	0.00018527	0.00560296
Switching of origins to a post-replicative state_Homo sapiens_R-HSA-69052	0.00018527	0.00560296
Respiratory electron transport_Homo sapiens_R-HSA-611105	0.00017578	0.00560296
APC/C:Cdh1 mediated degradation of Cdc20 and other APC/C:Cdh1 targeted proteins in late mitosis/early G1_Homo sapiens_R-HSA-174178	0.00020471	0.00561041
Cdc20:Phospho-APC/C mediated degradation of Cyclin A_Homo sapiens_R-HSA-174184	0.00020471	0.00561041
Respiratory electron transport, ATP synthesis by chemiosmotic coupling, and heat production by uncoupling proteins._Homo sapiens_R-HSA-163200	0.00019256	0.00561041
Removal of licensing factors from origins_Homo sapiens_R-HSA-69300	0.00022579	0.00582414
APC:Cdc20 mediated degradation of cell cycle proteins prior to satisfation of the cell cycle checkpoint_Homo sapiens_R-HSA-179419	0.00022579	0.00582414
mRNA Splicing - Major Pathway_Homo sapiens_R-HSA-72163	0.0002501	0.00626674

APC/C:Cdc20 mediated degradation of mitotic proteins_Homo sapiens_R-HSA-176409	0.0002733	0.00665794
Disease_Homo sapiens_R-HSA-1643685	0.00029218	0.00674504
Regulation of DNA replication_Homo sapiens_R-HSA-69304	0.00029995	0.00674504
Activation of APC/C and APC/C:Cdc20 mediated degradation of mitotic proteins_Homo sapiens_R-HSA-176814	0.00029995	0.00674504
Viral Messenger RNA Synthesis_Homo sapiens_R-HSA-168325	0.00035142	0.00770495
Transcriptional Regulation by TP53_Homo sapiens_R-HSA-3700989	0.0004222	0.00894991
Regulation of APC/C activators between G1/S and early anaphase_Homo sapiens_R-HSA-176408	0.00042862	0.00894991
mRNA Splicing_Homo sapiens_R-HSA-72172	0.00046467	0.00947717
Activation of the pre-replicative complex_Homo sapiens_R-HSA-68962	0.00053103	0.01058434
Antigen processing-Cross presentation_Homo sapiens_R-HSA-1236975	0.00055192	0.01075632
Hedgehog ligand biogenesis_Homo sapiens_R-HSA-5358346	0.00061142	0.01165681
Class I MHC mediated antigen processing & presentation_Homo sapiens_R-HSA-983169	0.00066372	0.01227819
ER-Phagosome pathway_Homo sapiens_R-HSA-1236974	0.00067201	0.01227819
APC/C-mediated degradation of cell cycle proteins_Homo sapiens_R-HSA-174143	0.00070245	0.01232098
Regulation of mitotic cell cycle_Homo sapiens_R-HSA-453276	0.00070245	0.01232098
Vpr-mediated nuclear import of PICs_Homo sapiens_R-HSA-180910	0.00072289	0.01243085
The citric acid (TCA) cycle and respiratory electron transport_Homo sapiens_R-HSA-1428517	0.00077284	0.01268543
Conversion from APC/C:Cdc20 to APC/C:Cdh1 in late anaphase_Homo sapiens_R-HSA-176407	0.00078109	0.01268543
Phosphorylation of the APC/C_Homo sapiens_R-HSA-176412	0.00078109	0.01268543
Cellular responses to stress_Homo sapiens_R-HSA-2262752	0.00080274	0.01280012
Complex I biogenesis_Homo sapiens_R-HSA-6799198	0.00081937	0.01283194
Rev-mediated nuclear export of HIV RNA_Homo sapiens_R-HSA-165054	0.00083623	0.01286616
Mitochondrial iron-sulfur cluster biogenesis_Homo sapiens_R-HSA-1362409	0.00088714	0.01341417
Diseases of signal transduction_Homo sapiens_R-HSA-5663202	0.00097957	0.01385621
Cell Cycle Checkpoints_Homo sapiens_R-HSA-69620	0.00097329	0.01385621
Inactivation of APC/C via direct inhibition of the APC/C complex_Homo sapiens_R-HSA-141430	0.00095894	0.01385621
Inhibition of the proteolytic activity of APC/C required for the onset of anaphase by mitotic spindle checkpoint components_Homo sapiens_R-HSA-141405	0.00095894	0.01385621
Interactions of Rev with host cellular proteins_Homo sapiens_R-HSA-177243	0.00110195	0.01510009
Interactions of Vpr with host cellular proteins_Homo sapiens_R-HSA-176033	0.00110195	0.01510009
Mitotic Spindle Checkpoint_Homo sapiens_R-HSA-69618	0.00116345	0.01569767
Hh mutants that don't undergo autocatalytic processing are degraded by ERAD_Homo sapiens_R-HSA-5362768	0.00151491	0.02012998
APC/C:Cdc20 mediated degradation of Cyclin B_Homo sapiens_R-HSA-174048	0.00166061	0.02173663
Hh mutants abrogate ligand secretion_Homo sapiens_R-HSA-5387390	0.00182545	0.02354296
Post-translational protein modification_Homo sapiens_R-HSA-597592	0.00202341	0.02535039
CDT1 association with the CDC6:ORC:origin complex_Homo sapiens_R-HSA-68827	0.0019977	0.02535039

APC-Cdc20 mediated degradation of Nek2A_Homo sapiens_R-HSA-179409	0.0022887	0.0278776
Post-translational modification: synthesis of GPI-anchored proteins_Homo sapiens_R-HSA-163125	0.0022887	0.0278776
Glucose transport_Homo sapiens_R-HSA-70153	0.00253517	0.03045676
Gap-filling DNA repair synthesis and ligation in TC-NER_Homo sapiens_R-HSA-6782210	0.00281201	0.03207343
Formation of HIV-1 elongation complex containing HIV-1 Tat_Homo sapiens_R-HSA-167200	0.00281603	0.03207343
HIV Transcription Elongation_Homo sapiens_R-HSA-167169	0.00281603	0.03207343
Tat-mediated elongation of the HIV-1 transcript_Homo sapiens_R-HSA-167246	0.00281603	0.03207343
SUMOylation of DNA replication proteins_Homo sapiens_R-HSA-4615885	0.00311871	0.03295306
Formation of HIV elongation complex in the absence of HIV Tat_Homo sapiens_R-HSA-167152	0.00311871	0.03295306
Formation of RNA Pol II elongation complex_Homo sapiens_R-HSA-112382	0.00311871	0.03295306
RNA Polymerase II Transcription Elongation_Homo sapiens_R-HSA-75955	0.00311871	0.03295306
RNA Pol II CTD phosphorylation and interaction with CE_Homo sapiens_R-HSA-167160	0.00306351	0.03295306
RNA Pol II CTD phosphorylation and interaction with CE_Homo sapiens_R-HSA-77075	0.00306351	0.03295306
SUMOylation of RNA binding proteins_Homo sapiens_R-HSA-4570464	0.00344414	0.03595851
Cellular Senescence_Homo sapiens_R-HSA-2559583	0.00386677	0.03989596
mRNA Capping_Homo sapiens_R-HSA-72086	0.00400031	0.04079383
Apoptosis_Homo sapiens_R-HSA-109581	0.00421922	0.04253164
Hexose transport_Homo sapiens_R-HSA-189200	0.00456636	0.04400767
NEP/NS2 Interacts with the Cellular Export Machinery_Homo sapiens_R-HSA-168333	0.00453404	0.04400767
Regulation of Glucokinase by Glucokinase Regulatory Protein_Homo sapiens_R-HSA-170822	0.00453404	0.04400767
Transport of Ribonucleoproteins into the Host Nucleus_Homo sapiens_R-HSA-168271	0.00453404	0.04400767
Programmed Cell Death_Homo sapiens_R-HSA-5357801	0.00479491	0.04570798
RNA Polymerase I, RNA Polymerase III, and Mitochondrial Transcription_Homo sapiens_R-HSA-504046	0.00505043	0.04762614
Export of Viral Ribonucleoproteins from Nucleus_Homo sapiens_R-HSA-168274	0.00511369	0.04770962
Metabolism of non-coding RNA_Homo sapiens_R-HSA-194441	0.00544534	0.04974545
snRNP Assembly_Homo sapiens_R-HSA-191859	0.00544534	0.04974545

Supplementary Table 5. List of differentially expressed genes in trisomy 21 versus normal embryos. Gene symbols, gene descriptions and average FPKM values, fold changes, P-values and adjusted P-values of genes differentially expressed in trisomy 21 versus normal embryos.

Gene Symbol	Log2 fold-change	pvalue	Adjusted-pvalue	Chromosome	Description
GREM1	4.96	8.80E-11	1.46E-06	chr15	gremlin 1, DAN family BMP antagonist [Source:HGNC Symbol;Acc:HGNC:2001]
FN1	4.28	1.05E-08	8.74E-05	chr2	fibronectin 1 [Source:HGNC Symbol;Acc:HGNC:3778]
GNLY	4.22	7.10E-08	0.000393194	chr2	granulysin [Source:HGNC Symbol;Acc:HGNC:4414]
B2M	3.89	1.99E-07	0.000824767	chr15	beta-2-microglobulin [Source:HGNC Symbol;Acc:HGNC:914]
IGFBP5	3.77	8.81E-07	0.002438159	chr2	insulin-like growth factor binding protein 5 [Source:HGNC Symbol;Acc:HGNC:5474]
HLA-B	3.58	1.93E-06	0.004007652	chr6	major histocompatibility complex, class I, B [Source:HGNC Symbol;Acc:HGNC:4932]
IGFBP7	3.43	3.69E-07	0.001226268	chr4	insulin-like growth factor binding protein 7 [Source:HGNC Symbol;Acc:HGNC:5476]
SCG5	3.33	1.83E-05	0.022191521	chr15	secretogranin V [Source:HGNC Symbol;Acc:HGNC:10816]
IFI27	3.31	3.35E-06	0.006175273	chr14	interferon, alpha-inducible protein 27 [Source:HGNC Symbol;Acc:HGNC:5397]
ZFPM2	3.17	5.33E-05	0.033533434	chr8	zinc finger protein, FOG family member 2 [Source:HGNC Symbol;Acc:HGNC:16700]
TCL1A	3.13	3.78E-05	0.029930957	chr14	T-cell leukemia/lymphoma 1A [Source:HGNC Symbol;Acc:HGNC:11648]
TIMP1	3.12	3.27E-05	0.027135326	chrX	TIMP metallopeptidase inhibitor 1 [Source:HGNC Symbol;Acc:HGNC:11820]
TMEM45A	3.00	2.68E-05	0.023422422	chr3	transmembrane protein 45A [Source:HGNC Symbol;Acc:HGNC:25480]
EMP3	2.97	0.000106072	0.046359222	chr19	epithelial membrane protein 3 [Source:HGNC Symbol;Acc:HGNC:3335]
MT1E	2.93	9.13E-05	0.042105669	chr16	metallothionein 1E [Source:HGNC Symbol;Acc:HGNC:7397]
HLA-A	2.89	9.78E-05	0.043911941	chr6	major histocompatibility complex, class I, A [Source:HGNC Symbol;Acc:HGNC:4931]
MTND6P4	2.58	1.90E-05	0.022191521	chr5	MT-ND6 pseudogene 4 [Source:HGNC Symbol;Acc:HGNC:39467]
RP11-632C17__A.1	2.56	2.22E-05	0.022191521	chr6	
THBS1	2.38	1.00E-05	0.016680788	chr15	thrombospondin 1 [Source:HGNC Symbol;Acc:HGNC:11785]
ARMC2	2.03	5.95E-05	0.034170245	chr6	armadillo repeat containing 2 [Source:HGNC Symbol;Acc:HGNC:23045]
HTRA1	1.91	8.88E-05	0.042105669	chr10	HtrA serine peptidase 1 [Source:HGNC Symbol;Acc:HGNC:9476]
DNAJC28	1.82	4.36E-05	0.031488519	chr21	DnaJ (Hsp40) homolog, subfamily C, member 28 [Source:HGNC Symbol;Acc:HGNC:1297]
CCNB1IP1	1.69	1.04E-06	0.002466107	chr14	cyclin B1 interacting protein 1, E3 ubiquitin protein ligase [Source:HGNC Symbol;Acc:HGNC:19437]
MZT2A	1.55	7.61E-05	0.03947812	chr2	mitotic spindle organizing protein 2A [Source:HGNC Symbol;Acc:HGNC:33187]
PKIG	1.44	2.40E-05	0.022191521	chr20	protein kinase (cAMP-dependent, catalytic) inhibitor gamma [Source:HGNC Symbol;Acc:HGNC:9019]

PBK	1.28	5.97E-05	0.034170245	chr8	PDZ binding kinase [Source:HGNC Symbol;Acc:HGNC:18282]
USP16	1.07	4.26E-05	0.031488519	chr21	ubiquitin specific peptidase 16 [Source:HGNC Symbol;Acc:HGNC:12614]
UBAP2L	-1.00	5.45E-05	0.033533434	chr1	ubiquitin associated protein 2-like [Source:HGNC Symbol;Acc:HGNC:29877]
CDC6	-1.06	5.14E-05	0.033533434	chr17	cell division cycle 6 [Source:HGNC Symbol;Acc:HGNC:1744]
KIAA0368	-1.14	8.21E-05	0.040111728	chr9	KIAA0368 [Source:HGNC Symbol;Acc:HGNC:29020]
NLN	-1.19	4.82E-05	0.033337875	chr5	neurolysin (metallopeptidase M3 family) [Source:HGNC Symbol;Acc:HGNC:16058]
RNF10	-1.31	6.80E-05	0.036404795	chr12	ring finger protein 10 [Source:HGNC Symbol;Acc:HGNC:10055]
SLC25A23	-1.34	2.22E-05	0.022191521	chr19	solute carrier family 25 (mitochondrial carrier; phosphate carrier), member 23 [Source:HGNC Symbol;Acc:HGNC:19375]
TRAF7	-1.65	2.41E-05	0.022191521	chr16	TNF receptor-associated factor 7, E3 ubiquitin protein ligase [Source:HGNC Symbol;Acc:HGNC:20456]
GRK6	-2.02	2.35E-05	0.022191521	chr5	G protein-coupled receptor kinase 6 [Source:HGNC Symbol;Acc:HGNC:4545]
RAB11FIP4	-2.05	1.93E-05	0.022191521	chr17	RAB11 family interacting protein 4 (class II) [Source:HGNC Symbol;Acc:HGNC:30267]
ANXA6	-2.53	7.96E-05	0.04004525	chr5	annexin A6 [Source:HGNC Symbol;Acc:HGNC:544]

Supplementary Table 6. List of enriched pathways (monosomies). P-values, adjusted P-values and enrichment scores of significantly enriched pathways in the list of genes that are frequently deregulated in monosomies.

Term	P-value	Adjusted P-value
Respiratory electron transport, ATP synthesis by chemiosmotic coupling, and heat production by uncoupling proteins._Homo sapiens_R-HSA-163200	3.75794E-38	4.62978E-35
Gene Expression_Homo sapiens_R-HSA-74160	2.10258E-36	1.29519E-33
The citric acid (TCA) cycle and respiratory electron transport_Homo sapiens_R-HSA-1428517	2.47654E-33	8.95797E-31
Respiratory electron transport_Homo sapiens_R-HSA-611105	2.90843E-33	8.95797E-31
Infectious disease_Homo sapiens_R-HSA-5663205	5.7498E-28	1.41675E-25
Metabolism of proteins_Homo sapiens_R-HSA-392499	6.4649E-23	1.32746E-20
Complex I biogenesis_Homo sapiens_R-HSA-6799198	2.60759E-21	4.58936E-19
HIV Infection_Homo sapiens_R-HSA-162906	2.64868E-20	4.07896E-18
Cell Cycle, Mitotic_Homo sapiens_R-HSA-69278	5.21818E-20	7.14311E-18
Translation_Homo sapiens_R-HSA-72766	8.04791E-19	9.91502E-17
Cell Cycle_Homo sapiens_R-HSA-1640170	3.17161E-18	3.5522E-16
Metabolism_Homo sapiens_R-HSA-1430728	3.73606E-18	3.83569E-16
rRNA processing_Homo sapiens_R-HSA-72312	2.26827E-17	2.14962E-15
Major pathway of rRNA processing in the nucleolus_Homo sapiens_R-HSA-6791226	2.75232E-17	2.34278E-15
SRP-dependent cotranslational protein targeting to membrane_Homo sapiens_R-HSA-1799339	2.85241E-17	2.34278E-15
G1/S Transition_Homo sapiens_R-HSA-69206	3.88858E-17	2.99421E-15
DNA Replication_Homo sapiens_R-HSA-69306	7.56708E-17	5.4839E-15
S Phase_Homo sapiens_R-HSA-69242	1.39151E-16	9.52411E-15
Assembly of the pre-replicative complex_Homo sapiens_R-HSA-68867	1.89362E-16	1.22787E-14
Regulation of DNA replication_Homo sapiens_R-HSA-69304	2.46628E-16	1.51923E-14
Removal of licensing factors from origins_Homo sapiens_R-HSA-69300	3.88132E-16	2.20109E-14
Mitotic G1-G1/S phases_Homo sapiens_R-HSA-453279	3.93052E-16	2.20109E-14
Synthesis of DNA_Homo sapiens_R-HSA-69239	5.91269E-16	3.16715E-14
M Phase_Homo sapiens_R-HSA-68886	9.20274E-16	4.72407E-14
DNA Replication Pre-Initiation_Homo sapiens_R-HSA-69002	1.19168E-15	5.64672E-14
M/G1 Transition_Homo sapiens_R-HSA-68874	1.19168E-15	5.64672E-14
Influenza Life Cycle_Homo sapiens_R-HSA-168255	1.97286E-15	9.00209E-14
Metabolism of amino acids and derivatives_Homo sapiens_R-HSA-71291	2.06462E-15	9.08431E-14
Mitochondrial translation elongation_Homo sapiens_R-HSA-5389840	3.10838E-15	1.27651E-13
Mitochondrial translation initiation_Homo sapiens_R-HSA-5368286	3.10838E-15	1.27651E-13
Mitotic Anaphase_Homo sapiens_R-HSA-68882	4.52766E-15	1.79938E-13
CDT1 association with the CDC6:ORC:origin complex_Homo sapiens_R-HSA-68827	5.70715E-15	2.19725E-13
Mitotic Metaphase and Anaphase_Homo sapiens_R-HSA-2555396	6.00246E-15	2.24092E-13
Mitochondrial translation_Homo sapiens_R-HSA-5368287	7.14857E-15	2.59031E-13
Orc1 removal from chromatin_Homo sapiens_R-HSA-68949	8.43789E-15	2.88763E-13
Switching of origins to a post-replicative state_Homo sapiens_R-HSA-69052	8.43789E-15	2.88763E-13
Influenza Infection_Homo sapiens_R-HSA-168254	1.45043E-14	4.82954E-13

Influenza Viral RNA Transcription and Replication_Homo sapiens_R-HSA-168273	1.58722E-14	5.14594E-13
Mitochondrial translation termination_Homo sapiens_R-HSA-5419276	2.08495E-14	6.58629E-13
Cyclin E associated events during G1/S transition_Homo sapiens_R-HSA-69202	2.29074E-14	7.05547E-13
GTP hydrolysis and joining of the 60S ribosomal subunit_Homo sapiens_R-HSA-72706	3.40537E-14	1.02327E-12
Cyclin A:Cdk2-associated events at S phase entry_Homo sapiens_R-HSA-69656	3.84311E-14	1.12731E-12
SCF-beta-TrCP mediated degradation of Emi1_Homo sapiens_R-HSA-174113	4.23717E-14	1.214E-12
Autodegradation of Cdh1 by Cdh1:APC/C_Homo sapiens_R-HSA-174084	5.80186E-14	1.62452E-12
APC/C:Cdc20 mediated degradation of mitotic proteins_Homo sapiens_R-HSA-176409	6.27798E-14	1.68141E-12
APC/C:Cdc20 mediated degradation of Securin_Homo sapiens_R-HSA-174154	6.21841E-14	1.68141E-12
Hh mutants that don't undergo autocatalytic processing are degraded by ERAD_Homo sapiens_R-HSA-5362768	7.76723E-14	2.036E-12
Cap-dependent Translation Initiation_Homo sapiens_R-HSA-72737	8.15499E-14	2.0504E-12
Eukaryotic Translation Initiation_Homo sapiens_R-HSA-72613	8.15499E-14	2.0504E-12
Regulation of APC/C activators between G1/S and early anaphase_Homo sapiens_R-HSA-176408	9.45933E-14	2.33078E-12
APC/C:Cdh1 mediated degradation of Cdc20 and other APC/C:Cdh1 targeted proteins in late mitosis/early G1_Homo sapiens_R-HSA-174178	1.04309E-13	2.4247E-12
Activation of APC/C and APC/C:Cdc20 mediated degradation of mitotic proteins_Homo sapiens_R-HSA-176814	1.0085E-13	2.4247E-12
Cdc20:Phospho-APC/C mediated degradation of Cyclin A_Homo sapiens_R-HSA-174184	1.04309E-13	2.4247E-12
3' -UTR-mediated translational regulation_Homo sapiens_R-HSA-157279	1.26452E-13	2.83252E-12
L13a-mediated translational silencing of Ceruloplasmin expression_Homo sapiens_R-HSA-156827	1.26452E-13	2.83252E-12
Degradation of DVL_Homo sapiens_R-HSA-4641258	1.39795E-13	3.0755E-12
Hedgehog ligand biogenesis_Homo sapiens_R-HSA-5358346	1.68791E-13	3.58798E-12
APC:Cdc20 mediated degradation of cell cycle proteins prior to satisfation of the cell cycle checkpoint_Homo sapiens_R-HSA-179419	1.68915E-13	3.58798E-12
APC/C-mediated degradation of cell cycle proteins_Homo sapiens_R-HSA-174143	2.03819E-13	4.18507E-12
Regulation of mitotic cell cycle_Homo sapiens_R-HSA-453276	2.03819E-13	4.18507E-12
Disease_Homo sapiens_R-HSA-1643685	2.41558E-13	4.87867E-12
Hh mutants abrogate ligand secretion_Homo sapiens_R-HSA-5387390	2.4727E-13	4.91349E-12
Separation of Sister Chromatids_Homo sapiens_R-HSA-2467813	2.56488E-13	5.01577E-12
Dectin-1 mediated noncanonical NF-kB signaling_Homo sapiens_R-HSA-5607761	2.70983E-13	5.21642E-12
AUF1 (hnRNP D0) binds and destabilizes mRNA_Homo sapiens_R-HSA-450408	3.81373E-13	7.22848E-12
NIK-->noncanonical NF-kB signaling_Homo sapiens_R-HSA-5676590	4.30215E-13	8.03068E-12
Selenocysteine synthesis_Homo sapiens_R-HSA-2408557	4.6527E-13	8.55541E-12
Vpu mediated degradation of CD4_Homo sapiens_R-HSA-180534	5.78118E-13	1.04741E-11
Selenoamino acid metabolism_Homo sapiens_R-HSA-2408522	7.32311E-13	1.30755E-11
Regulation of activated PAK-2p34 by proteasome mediated degradation_Homo sapiens_R-HSA-211733	1.57803E-12	2.77734E-11
Regulation of mRNA stability by proteins that bind AU-rich elements_Homo sapiens_R-HSA-450531	1.81101E-12	3.14248E-11
Vif-mediated degradation of APOBEC3G_Homo sapiens_R-HSA-180585	1.83893E-12	3.14661E-11
ER-Phagosome pathway_Homo sapiens_R-HSA-1236974	2.04232E-12	3.44677E-11
Formation of a pool of free 40S subunits_Homo sapiens_R-HSA-72689	2.61014E-12	4.34554E-11

Regulation of Apoptosis_Homo sapiens_R-HSA-169911	2.84123E-12	4.48768E-11
CDK-mediated phosphorylation and removal of Cdc6_Homo sapiens_R-HSA-69017	2.84123E-12	4.48768E-11
Ubiquitin-dependent degradation of Cyclin D1_Homo sapiens_R-HSA-69229	2.84123E-12	4.48768E-11
Ubiquitin-dependent degradation of Cyclin D_Homo sapiens_R-HSA-75815	2.84123E-12	4.48768E-11
HIV Life Cycle_Homo sapiens_R-HSA-162587	3.77871E-12	5.89287E-11
Viral mRNA Translation_Homo sapiens_R-HSA-192823	4.7102E-12	7.25372E-11
Autodegradation of the E3 ubiquitin ligase COP1_Homo sapiens_R-HSA-349425	5.01729E-12	7.63124E-11
Degradation of GLI2 by the proteasome_Homo sapiens_R-HSA-5610783	5.59998E-12	8.11668E-11
GLI3 is processed to GLI3R by the proteasome_Homo sapiens_R-HSA-5610785	5.59998E-12	8.11668E-11
Degradation of GLI1 by the proteasome_Homo sapiens_R-HSA-5610780	5.59998E-12	8.11668E-11
SCF(Skp2)-mediated degradation of p27/p21_Homo sapiens_R-HSA-187577	5.59998E-12	8.11668E-11
Nonsense Mediated Decay (NMD) independent of the Exon Junction Complex (EJC)_Homo sapiens_R-HSA-975956	5.70738E-12	8.17616E-11
p53-Independent DNA Damage Response_Homo sapiens_R-HSA-69610	8.69928E-12	1.20421E-10
p53-Independent G1/S DNA damage checkpoint_Homo sapiens_R-HSA-69613	8.69928E-12	1.20421E-10
Ubiquitin Mediated Degradation of Phosphorylated Cdc25A_Homo sapiens_R-HSA-69601	8.69928E-12	1.20421E-10
Host Interactions of HIV factors_Homo sapiens_R-HSA-162909	9.42899E-12	1.29072E-10
Eukaryotic Translation Termination_Homo sapiens_R-HSA-72764	1.46576E-11	1.98442E-10
Nonsense Mediated Decay (NMD) enhanced by the Exon Junction Complex (EJC)_Homo sapiens_R-HSA-975957	1.57821E-11	2.0907E-10
Nonsense-Mediated Decay (NMD)_Homo sapiens_R-HSA-927802	1.57821E-11	2.0907E-10
Apoptosis_Homo sapiens_R-HSA-109581	1.88994E-11	2.47702E-10
Activation of NF-kappaB in B cells_Homo sapiens_R-HSA-1169091	2.17469E-11	2.82022E-10
Degradation of AXIN_Homo sapiens_R-HSA-4641257	2.48524E-11	3.15651E-10
Stabilization of p53_Homo sapiens_R-HSA-69541	2.48524E-11	3.15651E-10
Peptide chain elongation_Homo sapiens_R-HSA-156902	2.60169E-11	3.27069E-10
Eukaryotic Translation Elongation_Homo sapiens_R-HSA-156842	3.01704E-11	3.75454E-10
CLEC7A (Dectin-1) signaling_Homo sapiens_R-HSA-5607764	3.67724E-11	4.53036E-10
Asymmetric localization of PCP proteins_Homo sapiens_R-HSA-4608870	3.71713E-11	4.53416E-10
Programmed Cell Death_Homo sapiens_R-HSA-5357801	3.85434E-11	4.65544E-10
Degradation of beta-catenin by the destruction complex_Homo sapiens_R-HSA-195253	6.67581E-11	7.98504E-10
Processing of Capped Intron-Containing Pre-mRNA_Homo sapiens_R-HSA-72203	1.08125E-10	1.28086E-09
Cross-presentation of soluble exogenous antigens (endosomes)_Homo sapiens_R-HSA-1236978	1.09742E-10	1.28764E-09
Late Phase of HIV Life Cycle_Homo sapiens_R-HSA-162599	1.21098E-10	1.40748E-09
tRNA processing_Homo sapiens_R-HSA-72306	1.30766E-10	1.50565E-09
Regulation of ornithine decarboxylase (ODC)_Homo sapiens_R-HSA-350562	1.83026E-10	2.08785E-09
The role of GTSE1 in G2/M progression after G2 checkpoint_Homo sapiens_R-HSA-8852276	2.64551E-10	2.99016E-09
Antigen processing-Cross presentation_Homo sapiens_R-HSA-1236975	3.46483E-10	3.88061E-09
MAPK6/MAPK4 signaling_Homo sapiens_R-HSA-5687128	4.06709E-10	4.5141E-09
mRNA Splicing - Major Pathway_Homo sapiens_R-HSA-72163	5.65334E-10	6.21867E-09
Ribosomal scanning and start codon recognition_Homo sapiens_R-HSA-72702	7.04544E-10	7.68141E-09
Regulation of RAS by GAPs_Homo sapiens_R-HSA-5658442	1.17267E-09	1.26731E-08
PCP/CE pathway_Homo sapiens_R-HSA-4086400	1.36983E-09	1.4675E-08

p53-Dependent G1 DNA Damage Response_Homo sapiens_R-HSA-69563	1.40334E-09	1.47771E-08
p53-Dependent G1/S DNA damage checkpoint_Homo sapiens_R-HSA-69580	1.40334E-09	1.47771E-08
Nucleotide Excision Repair_Homo sapiens_R-HSA-5696398	2.35962E-09	2.4636E-08
G1/S DNA Damage Checkpoints_Homo sapiens_R-HSA-69615	3.03445E-09	3.14155E-08
Metabolism of polyamines_Homo sapiens_R-HSA-351202	3.24869E-09	3.33532E-08
Cell Cycle Checkpoints_Homo sapiens_R-HSA-69620	3.78733E-09	3.85619E-08
Translation initiation complex formation_Homo sapiens_R-HSA-72649	4.3252E-09	4.36774E-08
Formation of TC-NER Pre-Incision Complex_Homo sapiens_R-HSA-6781823	4.97685E-09	4.98494E-08
Transcription-Coupled Nucleotide Excision Repair (TC-NER)_Homo sapiens_R-HSA-6781827	5.51311E-09	5.47754E-08
mRNA Splicing_Homo sapiens_R-HSA-72172	5.76516E-09	5.68214E-08
Activation of the mRNA upon binding of the cap-binding complex and eIFs, and subsequent binding to 43S_Homo sapiens_R-HSA-72662	6.45621E-09	6.31274E-08
C-type lectin receptors (CLRs)_Homo sapiens_R-HSA-5621481	7.2551E-09	7.03802E-08
Antigen processing: Ubiquitination & Proteasome degradation_Homo sapiens_R-HSA-983168	8.59533E-09	8.273E-08
Mitotic G2-G2/M phases_Homo sapiens_R-HSA-453274	9.30641E-09	8.88798E-08
G2/M Transition_Homo sapiens_R-HSA-69275	1.93344E-08	1.8323E-07
DNA Repair_Homo sapiens_R-HSA-73894	1.9849E-08	1.86672E-07
G2/M Checkpoints_Homo sapiens_R-HSA-69481	2.0394E-08	1.90344E-07
Class I MHC mediated antigen processing & presentation_Homo sapiens_R-HSA-983169	2.1078E-08	1.95249E-07
tRNA processing in the nucleus_Homo sapiens_R-HSA-6784531	2.47377E-08	2.27439E-07
Mitochondrial protein import_Homo sapiens_R-HSA-1268020	4.39049E-08	4.00672E-07
Formation of ATP by chemiosmotic coupling_Homo sapiens_R-HSA-163210	4.50288E-08	4.07908E-07
Formation of the ternary complex, and subsequently, the 43S complex_Homo sapiens_R-HSA-72695	5.24787E-08	4.71925E-07
Downstream TCR signaling_Homo sapiens_R-HSA-202424	5.35495E-08	4.78065E-07
Beta-catenin independent WNT signaling_Homo sapiens_R-HSA-3858494	6.17416E-08	5.47235E-07
Transcriptional Regulation by TP53_Homo sapiens_R-HSA-3700989	7.03898E-08	6.19431E-07
TNFR2 non-canonical NF-kB pathway_Homo sapiens_R-HSA-5668541	7.43031E-08	6.4923E-07
Hedgehog 'on' state_Homo sapiens_R-HSA-5632684	8.29134E-08	7.19361E-07
Gap-filling DNA repair synthesis and ligation in TC-NER_Homo sapiens_R-HSA-6782210	1.44495E-07	1.24488E-06
Global Genome Nucleotide Excision Repair (GG-NER)_Homo sapiens_R-HSA-5696399	1.49253E-07	1.27694E-06
Signaling by Insulin receptor_Homo sapiens_R-HSA-74752	5.35416E-07	4.54919E-06
Cellular responses to stress_Homo sapiens_R-HSA-2262752	5.83106E-07	4.92045E-06
Membrane Trafficking_Homo sapiens_R-HSA-199991	8.29232E-07	6.94976E-06
FCERI mediated NF-kB activation_Homo sapiens_R-HSA-2871837	8.46521E-07	7.04671E-06
HIV Transcription Initiation_Homo sapiens_R-HSA-167161	9.40423E-07	7.52339E-06
RNA Polymerase II HIV Promoter Escape_Homo sapiens_R-HSA-167162	9.40423E-07	7.52339E-06
RNA Polymerase II Promoter Escape_Homo sapiens_R-HSA-73776	9.40423E-07	7.52339E-06
RNA Polymerase II Transcription Initiation And Promoter Clearance_Homo sapiens_R-HSA-76042	9.40423E-07	7.52339E-06
RNA Polymerase II Transcription Initiation_Homo sapiens_R-HSA-75953	9.40423E-07	7.52339E-06
RNA Polymerase II Transcription Pre-Initiation And Promoter Opening_Homo sapiens_R-HSA-73779	9.40423E-07	7.52339E-06
TP53 Regulates Metabolic Genes_Homo sapiens_R-HSA-5628897	9.83113E-07	7.81416E-06

TCR signaling_Homo sapiens_R-HSA-202403	1.09371E-06	8.63749E-06
Cytosolic tRNA aminoacylation_Homo sapiens_R-HSA-379716	1.4335E-06	1.12489E-05
Formation of the Early Elongation Complex_Homo sapiens_R-HSA-113418	2.29565E-06	1.77877E-05
Formation of the HIV-1 Early Elongation Complex_Homo sapiens_R-HSA-167158	2.29565E-06	1.77877E-05
Hedgehog 'off' state_Homo sapiens_R-HSA-5610787	2.43494E-06	1.87491E-05
Transferrin endocytosis and recycling_Homo sapiens_R-HSA-917977	2.49067E-06	1.9059E-05
Organelle biogenesis and maintenance_Homo sapiens_R-HSA-1852241	2.78627E-06	2.11894E-05
TCF dependent signaling in response to WNT_Homo sapiens_R-HSA-201681	3.3844E-06	2.54553E-05
Signaling by Hedgehog_Homo sapiens_R-HSA-5358351	3.38853E-06	2.54553E-05
Dual incision in TC-NER_Homo sapiens_R-HSA-6782135	3.90478E-06	2.91557E-05
Diseases of signal transduction_Homo sapiens_R-HSA-5663202	4.14953E-06	3.07965E-05
Insulin receptor recycling_Homo sapiens_R-HSA-77387	4.18536E-06	3.08765E-05
Post-translational protein modification_Homo sapiens_R-HSA-597592	4.8849E-06	3.58226E-05
RNA Pol II CTD phosphorylation and interaction with CE_Homo sapiens_R-HSA-167160	6.81243E-06	4.93701E-05
RNA Pol II CTD phosphorylation and interaction with CE_Homo sapiens_R-HSA-77075	6.81243E-06	4.93701E-05
RNA Polymerase I, RNA Polymerase III, and Mitochondrial Transcription_Homo sapiens_R-HSA-504046	6.97247E-06	5.02344E-05
Metabolism of non-coding RNA_Homo sapiens_R-HSA-194441	7.2851E-06	5.188E-05
snRNP Assembly_Homo sapiens_R-HSA-191859	7.2851E-06	5.188E-05
RNA Polymerase II Transcription_Homo sapiens_R-HSA-73857	9.87017E-06	6.98853E-05
tRNA Aminoacylation_Homo sapiens_R-HSA-379724	1.2666E-05	8.9169E-05
Asparagine N-linked glycosylation_Homo sapiens_R-HSA-446203	1.62672E-05	0.00011387
mRNA Capping_Homo sapiens_R-HSA-72086	1.66334E-05	0.000115776
Iron uptake and transport_Homo sapiens_R-HSA-917937	1.75685E-05	0.000121598
Signaling by Wnt_Homo sapiens_R-HSA-195721	1.79925E-05	0.000123837
Unfolded Protein Response (UPR)_Homo sapiens_R-HSA-381119	1.87859E-05	0.000128579
RNA Polymerase II Pre-transcription Events_Homo sapiens_R-HSA-674695	1.99421E-05	0.000135738
RNA Polymerase I Promoter Escape_Homo sapiens_R-HSA-73772	2.50743E-05	0.000169734
ER to Golgi Anterograde Transport_Homo sapiens_R-HSA-199977	3.07929E-05	0.000207305
Synthesis of diphthamide-EEF2_Homo sapiens_R-HSA-5358493	3.12395E-05	0.000209169
Mitophagy_Homo sapiens_R-HSA-5205647	3.18862E-05	0.000210074
Pink/Parkin Mediated Mitophagy_Homo sapiens_R-HSA-5205685	3.18862E-05	0.000210074
mTORC1-mediated signalling_Homo sapiens_R-HSA-166208	3.18862E-05	0.000210074
COPI-mediated anterograde transport_Homo sapiens_R-HSA-6807878	3.83396E-05	0.000251247
Transcription of the HIV genome_Homo sapiens_R-HSA-167172	4.05676E-05	0.000263049
Macroautophagy_Homo sapiens_R-HSA-1632852	4.05676E-05	0.000263049
Association of licensing factors with the pre-replicative complex_Homo sapiens_R-HSA-69298	4.82277E-05	0.000311081
RNA Polymerase I Transcription Termination_Homo sapiens_R-HSA-73863	5.35622E-05	0.000343691
Chromosome Maintenance_Homo sapiens_R-HSA-73886	5.92977E-05	0.000378522
Endosomal Sorting Complex Required For Transport (ESCRT)_Homo sapiens_R-HSA-917729	6.66244E-05	0.0004231
Formation of Incision Complex in GG-NER_Homo sapiens_R-HSA-5696395	7.97146E-05	0.000493497
Formation of HIV-1 elongation complex containing HIV-1 Tat_Homo sapiens_R-HSA-167200	7.97146E-05	0.000493497
HIV Transcription Elongation_Homo sapiens_R-HSA-167169	7.97146E-05	0.000493497

Tat-mediated elongation of the HIV-1 transcript_Homo sapiens_R-HSA-167246	7.97146E-05	0.000493497
Cyclin D associated events in G1_Homo sapiens_R-HSA-69231	8.01131E-05	0.000493497
G1 Phase_Homo sapiens_R-HSA-69236	8.01131E-05	0.000493497
Vesicle-mediated transport_Homo sapiens_R-HSA-5653656	8.73798E-05	0.000535582
Intra-Golgi and retrograde Golgi-to-ER traffic_Homo sapiens_R-HSA-6811442	0.00010021	0.000611181
Formation of HIV elongation complex in the absence of HIV Tat_Homo sapiens_R-HSA-167152	0.000105679	0.000635104
Formation of RNA Pol II elongation complex_Homo sapiens_R-HSA-112382	0.000105679	0.000635104
RNA Polymerase II Transcription Elongation_Homo sapiens_R-HSA-75955	0.000105679	0.000635104
ROS, RNS production in response to bacteria_Homo sapiens_R-HSA-1222556	0.000106477	0.000636792
tRNA modification in the nucleus and cytosol_Homo sapiens_R-HSA-6782315	0.000108671	0.000646775
Budding and maturation of HIV virion_Homo sapiens_R-HSA-162588	0.000119829	0.000709759
RNA Polymerase I Chain Elongation_Homo sapiens_R-HSA-73777	0.000183998	0.001084622
RNA Polymerase III Abortive And Retractive Initiation_Homo sapiens_R-HSA-749476	0.000192828	0.001125899
RNA Polymerase III Transcription_Homo sapiens_R-HSA-74158	0.000192828	0.001125899
RNA Polymerase III Transcription Initiation_Homo sapiens_R-HSA-76046	0.000199012	0.001156523
Downstream signaling events of B Cell Receptor (BCR)_Homo sapiens_R-HSA-1168372	0.000201379	0.001164786
Insulin receptor signalling cascade_Homo sapiens_R-HSA-74751	0.000242449	0.001395783
Viral Messenger RNA Synthesis_Homo sapiens_R-HSA-168325	0.000252605	0.001447485
Prefoldin mediated transfer of substrate to CCT/TriC_Homo sapiens_R-HSA-389957	0.000253939	0.001448392
Negative epigenetic regulation of rRNA expression_Homo sapiens_R-HSA-5250941	0.000255152	0.001448605
XBP1(S) activates chaperone genes_Homo sapiens_R-HSA-381038	0.000262676	0.001484482
DNA strand elongation_Homo sapiens_R-HSA-69190	0.000269037	0.001513488
Transport to the Golgi and subsequent modification_Homo sapiens_R-HSA-948021	0.000332586	0.001862481
IRS-mediated signalling_Homo sapiens_R-HSA-112399	0.000350757	0.00195535
IRE1alpha activates chaperones_Homo sapiens_R-HSA-381070	0.000408552	0.002267277
NRIF signals cell death from the nucleus_Homo sapiens_R-HSA-205043	0.000422966	0.002336746
mTOR signalling_Homo sapiens_R-HSA-165159	0.00046103	0.002535667
IGF1R signaling cascade_Homo sapiens_R-HSA-2428924	0.000487377	0.002645148
IRS-related events triggered by IGF1R_Homo sapiens_R-HSA-2428928	0.000487377	0.002645148
Signaling by Type 1 Insulin-like Growth Factor 1 Receptor (IGF1R)_Homo sapiens_R-HSA-2404192	0.000487377	0.002645148
COPII (Coat Protein 2) Mediated Vesicle Transport_Homo sapiens_R-HSA-204005	0.000529134	0.002859182
Nuclear Envelope Breakdown_Homo sapiens_R-HSA-2980766	0.000587032	0.00315818
PKB-mediated events_Homo sapiens_R-HSA-109703	0.000596215	0.003193639
Mitotic Prophase_Homo sapiens_R-HSA-68875	0.000649275	0.003447872
Mitotic Prometaphase_Homo sapiens_R-HSA-68877	0.000649275	0.003447872
APC-Cdc20 mediated degradation of Nek2A_Homo sapiens_R-HSA-179409	0.000659568	0.003487502
Activation of the pre-replicative complex_Homo sapiens_R-HSA-68962	0.000673462	0.00354575
RHO GTPases Activate Formins_Homo sapiens_R-HSA-5663220	0.000687606	0.00360481
mRNA Splicing - Minor Pathway_Homo sapiens_R-HSA-72165	0.000726532	0.003792743
RNA Polymerase I Transcription Initiation_Homo sapiens_R-HSA-73762	0.00084418	0.004388309
Transcriptional regulation by small RNAs_Homo sapiens_R-HSA-5578749	0.00106352	0.005505279
Cooperation of Prefoldin and TriC/CCT in actin and tubulin folding_Homo sapiens_R-HSA-389958	0.001188717	0.006109323

Assembly Of The HIV Virion_Homo sapiens_R-HSA-175474	0.001200046	0.006109323
Initiation of Nuclear Envelope Reformation_Homo sapiens_R-HSA-2995383	0.001200046	0.006109323
Nuclear Envelope Reassembly_Homo sapiens_R-HSA-2995410	0.001200046	0.006109323
RHO GTPase Effectors_Homo sapiens_R-HSA-195258	0.001250026	0.006299582
NoRC negatively regulates rRNA expression_Homo sapiens_R-HSA-427413	0.001252758	0.006299582
Signaling by TGF-beta Receptor Complex_Homo sapiens_R-HSA-170834	0.001252758	0.006299582
Signaling by the B Cell Receptor (BCR)_Homo sapiens_R-HSA-983705	0.001283593	0.006428402
Resolution of Sister Chromatid Cohesion_Homo sapiens_R-HSA-2500257	0.001305372	0.006511003
TP53 Regulates Transcription of DNA Repair Genes_Homo sapiens_R-HSA-6796648	0.001322692	0.006570795
DNA Damage Recognition in GG-NER_Homo sapiens_R-HSA-5696394	0.001386653	0.006860868
Signalling by NGF_Homo sapiens_R-HSA-166520	0.001402984	0.006913905
Rev-mediated nuclear export of HIV RNA_Homo sapiens_R-HSA-165054	0.001546645	0.007561374
E2F mediated regulation of DNA replication_Homo sapiens_R-HSA-113510	0.001546645	0.007561374
RNA Polymerase III Transcription Initiation From Type 1 Promoter_Homo sapiens_R-HSA-76061	0.001666697	0.008116088
Gene Silencing by RNA_Homo sapiens_R-HSA-211000	0.001700181	0.008150283
Protein folding_Homo sapiens_R-HSA-391251	0.001700181	0.008150283
Abortive elongation of HIV-1 transcript in the absence of Tat_Homo sapiens_R-HSA-167242	0.001699822	0.008150283
MicroRNA (miRNA) biogenesis_Homo sapiens_R-HSA-203927	0.001699822	0.008150283
Gamma carboxylation, hypusine formation and arylsulfatase activation_Homo sapiens_R-HSA-163841	0.00175029	0.008357974
Epigenetic regulation of gene expression_Homo sapiens_R-HSA-212165	0.001812978	0.008623895
SUMOylation of RNA binding proteins_Homo sapiens_R-HSA-4570464	0.00186853	0.008853957
RNA Polymerase I Promoter Clearance_Homo sapiens_R-HSA-73854	0.001999614	0.009438792
Regulated proteolysis of p75NTR_Homo sapiens_R-HSA-193692	0.002051098	0.009644857
Golgi-to-ER retrograde transport_Homo sapiens_R-HSA-8856688	0.002252064	0.010544572
Translesion Synthesis by POLH_Homo sapiens_R-HSA-110320	0.00226811	0.010544572
Citric acid cycle (TCA cycle)_Homo sapiens_R-HSA-71403	0.00226811	0.010544572
DNA Double-Strand Break Repair_Homo sapiens_R-HSA-5693532	0.002304744	0.010622911
RNA polymerase II transcribes snRNA genes_Homo sapiens_R-HSA-6807505	0.002319451	0.010622911
Deposition of new CENPA-containing nucleosomes at the centromere_Homo sapiens_R-HSA-606279	0.002315048	0.010622911
Nucleosome assembly_Homo sapiens_R-HSA-774815	0.002315048	0.010622911
Interactions of Rev with host cellular proteins_Homo sapiens_R-HSA-177243	0.002523478	0.011514536
NEP/NS2 Interacts with the Cellular Export Machinery_Homo sapiens_R-HSA-168333	0.002845422	0.012935645
Processive synthesis on the lagging strand_Homo sapiens_R-HSA-69183	0.002908045	0.013171735
RNA Polymerase I Transcription_Homo sapiens_R-HSA-73864	0.003087962	0.013884559
Glucose metabolism_Homo sapiens_R-HSA-70326	0.003087962	0.013884559
Inactivation of APC/C via direct inhibition of the APC/C complex_Homo sapiens_R-HSA-141430	0.003175773	0.014175912
Inhibition of the proteolytic activity of APC/C required for the onset of anaphase by mitotic spindle checkpoint components_Homo sapiens_R-HSA-141405	0.003175773	0.014175912
MAPK family signaling cascades_Homo sapiens_R-HSA-5683057	0.003432859	0.015118395
Membrane binding and targetting of GAG proteins_Homo sapiens_R-HSA-174490	0.003435999	0.015118395
Synthesis And Processing Of GAG, GAGPOL Polyproteins_Homo sapiens_R-HSA-174495	0.003435999	0.015118395

Processive synthesis on the C-strand of the telomere_Homo sapiens_R-HSA-174414	0.003435999	0.015118395
Export of Viral Ribonucleoproteins from Nucleus_Homo sapiens_R-HSA-168274	0.003640738	0.015962237
Signalling to p38 via RIT and RIN_Homo sapiens_R-HSA-187706	0.003697687	0.016154436
Transport of Mature Transcript to Cytoplasm_Homo sapiens_R-HSA-72202	0.003834851	0.016694475
COPI-dependent Golgi-to-ER retrograde traffic_Homo sapiens_R-HSA-6811434	0.004056324	0.017596448
Transport of Mature mRNA derived from an Intron-Containing Transcript_Homo sapiens_R-HSA-159236	0.004121636	0.017817037
Chaperonin-mediated protein folding_Homo sapiens_R-HSA-390466	0.004336372	0.01861467
Mitotic Spindle Checkpoint_Homo sapiens_R-HSA-69618	0.004336342	0.01861467
Signaling by Rho GTPases_Homo sapiens_R-HSA-194315	0.004517689	0.019325671
Adaptive Immune System_Homo sapiens_R-HSA-1280218	0.004548303	0.019389304
GRB2 events in EGFR signaling_Homo sapiens_R-HSA-179812	0.004815374	0.020110309
RAF/MAP kinase cascade_Homo sapiens_R-HSA-5673001	0.004815374	0.020110309
SHC1 events in EGFR signaling_Homo sapiens_R-HSA-180336	0.004815374	0.020110309
SHC1 events in ERBB4 signaling_Homo sapiens_R-HSA-1250347	0.004815374	0.020110309
SOS-mediated signalling_Homo sapiens_R-HSA-112412	0.004815374	0.020110309
Deadenylation-dependent mRNA decay_Homo sapiens_R-HSA-429914	0.004813832	0.020110309
Transcriptional activity of SMAD2/SMAD3:SMAD4 heterotrimer_Homo sapiens_R-HSA-2173793	0.004903353	0.020339836
SUMOylation of DNA replication proteins_Homo sapiens_R-HSA-4615885	0.004903353	0.020339836
Signaling by Leptin_Homo sapiens_R-HSA-2586552	0.004930737	0.020384793
FRS-mediated FGFR1 signaling_Homo sapiens_R-HSA-5654693	0.005172339	0.021100404
FRS-mediated FGFR2 signaling_Homo sapiens_R-HSA-5654700	0.005172339	0.021100404
FRS-mediated FGFR3 signaling_Homo sapiens_R-HSA-5654706	0.005172339	0.021100404
FRS-mediated FGFR4 signaling_Homo sapiens_R-HSA-5654712	0.005172339	0.021100404
RNA Polymerase III Transcription Initiation From Type 2 Promoter_Homo sapiens_R-HSA-76066	0.005254478	0.021364744
Signaling by ERBB4_Homo sapiens_R-HSA-1236394	0.005292487	0.0214485
Unwinding of DNA_Homo sapiens_R-HSA-176974	0.005383746	0.021746804
Interleukin receptor SHC signaling_Homo sapiens_R-HSA-912526	0.005667833	0.022819509
Transport of the SLBP independent Mature mRNA_Homo sapiens_R-HSA-159227	0.005745316	0.022981265
Cargo concentration in the ER_Homo sapiens_R-HSA-5694530	0.005745316	0.022981265
Signalling to ERKs_Homo sapiens_R-HSA-187687	0.005772938	0.023012967
Cyclin A/B1 associated events during G2/M transition_Homo sapiens_R-HSA-69273	0.0057906	0.023012967
Dual Incision in GG-NER_Homo sapiens_R-HSA-5696400	0.005923155	0.023464073
G1/S-Specific Transcription_Homo sapiens_R-HSA-69205	0.005979897	0.023612926
Insulin-like Growth Factor-2 mRNA Binding Proteins (IGF2BPs/IMPs/VICKZs) bind RNA_Homo sapiens_R-HSA-428359	0.006032776	0.023745623
Signalling to RAS_Homo sapiens_R-HSA-167044	0.006069938	0.023815808
Signaling by SCF-KIT_Homo sapiens_R-HSA-1433557	0.006289085	0.02459731
rRNA modification in the nucleus_Homo sapiens_R-HSA-6790901	0.006345504	0.024739432
ARMS-mediated activation_Homo sapiens_R-HSA-170984	0.00638018	0.024796156
NGF signalling via TRKA from the plasma membrane_Homo sapiens_R-HSA-187037	0.006660366	0.025803682
RNA Polymerase III Transcription Initiation From Type 3 Promoter_Homo sapiens_R-HSA-76071	0.006689591	0.025835662
Frs2-mediated activation_Homo sapiens_R-HSA-170968	0.006832049	0.026303387
VEGFR2 mediated cell proliferation_Homo sapiens_R-HSA-5218921	0.006946459	0.026660553

DNA Damage Bypass_Homo sapiens_R-HSA-73893	0.007015186	0.026840711
Transport of the SLBP Dependant Mature mRNA_Homo sapiens_R-HSA-159230	0.007098244	0.027074419
MAPK1/MAPK3 signaling_Homo sapiens_R-HSA-5684996	0.007310422	0.027797653
Telomere Maintenance_Homo sapiens_R-HSA-157579	0.007373009	0.027949375
Transcriptional regulation of white adipocyte differentiation_Homo sapiens_R-HSA-381340	0.007447892	0.028146635
APC/C:Cdc20 mediated degradation of Cyclin B_Homo sapiens_R-HSA-174048	0.007580316	0.028472407
Gap-filling DNA repair synthesis and ligation in GG-NER_Homo sapiens_R-HSA-5696397	0.007580316	0.028472407
Prolonged ERK activation events_Homo sapiens_R-HSA-169893	0.007816468	0.029270177
Signaling by FGFR2_Homo sapiens_R-HSA-5654738	0.007870117	0.029381772
TP53 Regulates Transcription of Genes Involved in G2 Cell Cycle Arrest_Homo sapiens_R-HSA-6804114	0.008182716	0.030456514
Lysosome Vesicle Biogenesis_Homo sapiens_R-HSA-432720	0.008681818	0.032216865
Signaling by EGFR_Homo sapiens_R-HSA-177929	0.008788192	0.032513673
Interleukin-2 signaling_Homo sapiens_R-HSA-451927	0.009019545	0.033269698
Pyruvate metabolism and Citric Acid (TCA) cycle_Homo sapiens_R-HSA-71406	0.009777102	0.035956386
Cellular Senescence_Homo sapiens_R-HSA-2559583	0.009856759	0.03609047
SUMO is conjugated to E1 (UBA2:SAE1)_Homo sapiens_R-HSA-3065676	0.00987215	0.03609047
PTK6 Regulates RTKs and Their Effectors AKT1 and DOK1_Homo sapiens_R-HSA-8849469	0.00996086	0.036199941
Pentose phosphate pathway (hexose monophosphate shunt)_Homo sapiens_R-HSA-71336	0.00996086	0.036199941
Signaling by FGFR_Homo sapiens_R-HSA-190236	0.010237867	0.037051223
Intrinsic Pathway for Apoptosis_Homo sapiens_R-HSA-109606	0.010255249	0.037051223
MHC class II antigen presentation_Homo sapiens_R-HSA-2132295	0.010535768	0.037953409
Cellular response to heat stress_Homo sapiens_R-HSA-3371556	0.010659256	0.038286306
Conversion from APC/C:Cdc20 to APC/C:Cdh1 in late anaphase_Homo sapiens_R-HSA-176407	0.010912534	0.038968818
Signaling by FGFR2 IIIa TM_Homo sapiens_R-HSA-8851708	0.010912534	0.038968818
Early Phase of HIV Life Cycle_Homo sapiens_R-HSA-162594	0.011378862	0.040283787
Mismatch repair (MMR) directed by MSH2:MSH6 (MutSalpha)_Homo sapiens_R-HSA-5358565	0.011378862	0.040283787
Removal of the Flap Intermediate_Homo sapiens_R-HSA-69166	0.011378862	0.040283787
Post-translational modification: synthesis of GPI-anchored proteins_Homo sapiens_R-HSA-163125	0.012333791	0.043539342
Translesion synthesis by Y family DNA polymerases bypasses lesions on DNA template_Homo sapiens_R-HSA-110313	0.012633629	0.044217701
Transport of Mature mRNA Derived from an Intronless Transcript_Homo sapiens_R-HSA-159231	0.012633629	0.044217701
Apoptotic cleavage of cellular proteins_Homo sapiens_R-HSA-111465	0.012633629	0.044217701
Pausing and recovery of Tat-mediated HIV elongation_Homo sapiens_R-HSA-167238	0.012781952	0.044484081
Tat-mediated HIV elongation arrest and recovery_Homo sapiens_R-HSA-167243	0.012781952	0.044484081
Clathrin derived vesicle budding_Homo sapiens_R-HSA-421837	0.013202083	0.045688106
trans-Golgi Network Vesicle Budding_Homo sapiens_R-HSA-199992	0.013202083	0.045688106
Intra-Golgi traffic_Homo sapiens_R-HSA-6811438	0.014260512	0.048938582
Synthesis, secretion, and inactivation of Glucagon-like Peptide-1 (GLP-1)_Homo sapiens_R-HSA-381771	0.014228289	0.048938582

Lagging Strand Synthesis_Homo sapiens_R-HSA-69186	0.014228289	0.048938582
